# Introducing urea into tirapazamine derivatives to enhance anticancer therapy

**DOI:** 10.1093/nsr/nwae038

**Published:** 2024-02-05

**Authors:** Yajun Xu, Jianlin Lv, Chaoying Kong, Ya Liu, Kun Wang, Zhaohui Tang, Xuesi Chen

**Affiliations:** Key Laboratory of Polymer Ecomaterials, Changchun Institute of Applied Chemistry, Chinese Academy of Sciences, Changchun 130022, China; Key Laboratory of Polymer Ecomaterials, Changchun Institute of Applied Chemistry, Chinese Academy of Sciences, Changchun 130022, China; School of Applied Chemistry and Engineering, University of Science and Technology of China, Hefei 230026, China; Key Laboratory of Polymer Ecomaterials, Changchun Institute of Applied Chemistry, Chinese Academy of Sciences, Changchun 130022, China; School of Applied Chemistry and Engineering, University of Science and Technology of China, Hefei 230026, China; Key Laboratory of Polymer Ecomaterials, Changchun Institute of Applied Chemistry, Chinese Academy of Sciences, Changchun 130022, China; School of Applied Chemistry and Engineering, University of Science and Technology of China, Hefei 230026, China; Key Laboratory of Polymer Ecomaterials, Changchun Institute of Applied Chemistry, Chinese Academy of Sciences, Changchun 130022, China; Key Laboratory of Polymer Ecomaterials, Changchun Institute of Applied Chemistry, Chinese Academy of Sciences, Changchun 130022, China; School of Applied Chemistry and Engineering, University of Science and Technology of China, Hefei 230026, China; Key Laboratory of Polymer Ecomaterials, Changchun Institute of Applied Chemistry, Chinese Academy of Sciences, Changchun 130022, China; School of Applied Chemistry and Engineering, University of Science and Technology of China, Hefei 230026, China

**Keywords:** tirapazamine, vascular disrupting agent, hypoxia-activated prodrug, combretastatin A4, urea

## Abstract

Tirapazamine (TPZ) has been approved for multiple clinical trials relying on its excellent anticancer potential. However, as a typical hypoxia-activated prodrug (HAP), TPZ did not exhibit survival advantages in Phase III clinical trials when used in combination therapy due to the insufficient hypoxia levels in patients’ tumors. In this study, to improve the therapeutic effects of TPZ, we first introduced urea to synthesize a series of urea-containing derivatives of TPZ. All urea-containing TPZ derivatives showed increased hypoxic cytotoxicity (9.51–30.85-fold) compared with TPZ, while maintaining hypoxic selectivity. TPZP, one of these derivatives, showed 20-fold higher cytotoxicity than TPZ while maintaining a similar hypoxic cytotoxicity ratio. To highly efficiently deliver TPZP to the tumors and reduce its side effects on healthy tissues, we further prepared TPZP into a nanodrug with fibrin-targeting ability: FT11-TPZP-NPs. CA4-NPs, a vascular disrupting agent, was used to increase the fibrin level within tumors and exacerbate tumor hypoxia. By being combined with CA4-NPs, FT11-TPZP-NPs can accumulate in the hypoxia-aggravated tumors and activate sufficiently to kill tumor cells. After a single-dose treatment, FT11-TPZP-NPs + CA4-NPs showed a high inhibition rate of 98.1% against CT26 tumor models with an initial volume of ∼480 mm^3^ and four out of six tumors were completely eliminated; it thereby exerted a significant antitumor effect. This study provides a new strategy for improving the therapeutic effect of TPZ and other HAPs in anticancer therapy.

## Teaser

Urea-containing derivatives of tirapazamine exhibited enhanced hypoxic cytotoxicity while preserving hypoxic selectivity, and its nanodrug with fibrin-targeting ability achieved effective tumor suppression by combining treatment with a vascular disrupting agent.

## INTRODUCTION

Hypoxia is a common feature of solid tumors and is regarded as one of the best therapeutic targets for cancer treatment [1,2]. As bioreductive activation drugs, hypoxia-activated prodrugs (HAPs) can be selectively activated into highly cytotoxic species under hypoxic conditions and kill the hypoxic cells of solid tumors [3,4]. Tirapazamine (TPZ) is a typical HAP that exhibits selective higher cytotoxicity to hypoxic tumor cells and lower cytotoxicity to normal tissue [5,6]. It is universally agreed that the hypoxic selectivity of TPZ arises from the initial one-electron reduction process of TPZ to form the TPZ radical anion [7,8]. The TPZ radical anion can be oxidized to TPZ by molecular oxygen, preventing the formation of a subsequent TPZ-derived radical that induces DNA damage [9,10]. Until now, the US Food and Drug Administration (FDA) has approved ∼17 clinical trials related to TPZ. Although TPZ showed encouraging results in early clinical studies, Phase III clinical trials failed to improve overall survival. A subsequent clinical trial has shown that the limited clinical activity of TPZ was due to the fact that the tumors of patients were not hypoxic enough to efficiently activate a sufficient concentration of TPZ [11]. Therefore, the therapeutic effect of TPZ can be enhanced by selectively improving the hypoxia level of tumors to activate a sufficient concentration of TPZ. Another approach is to increase the cytotoxicity of TPZ to tumor cells.

There are various pathways that can increase hypoxia levels in tumors. For example, sonodynamic therapy (SDT) [12,13] or photodynamic therapy (PDT) [14,15] can increase the hypoxic levels by enhancing oxygen consumption in tumors. On the other hand, transcatheter hepatic artery embolization (TACE) can induce vascular occlusion in tumors, causing an increase in hypoxia [16]. However, SDT, PDT and TACE, as local therapies, are unsuitable for the treatment of tumors with metastatic lesions [17]. Tumor blood vessels supply the necessary oxygen and nutrients for the tumor growth. Vascular disrupting agents (VDAs) can effectively shut down the established tumor blood vessels, causing a significant reduction in oxygen and nutrient supply to solid tumors [18–20]. VDAs not only result in secondary necrosis of tumor cells, but also trigger coagulation cascade reactions and increase hypoxia levels in tumors [21–23]. Based on combretastatin A4 (CA4), a representative VDA, we have developed a nanosized VDA called CA4-NPs [24]. CA4-NPs can selectively disrupt tumor blood vessels, leading to hemorrhage and increased hypoxia within the treated tumors. The hemorrhaging triggers a coagulation cascade reaction. In the beginning, platelets quickly form an unstable plug at the site of bleeding to achieve primary hemostasis. Afterwards, the activation of coagulation factors triggers a chain reaction that converts fibrinogen into fibrin, which further cross-links to stabilize the clot and reduce excessive blood loss. In other words, the administration of CA4-NPs can not only exacerbate tumor hypoxia, but also lead to a significant accumulation of fibrin within the tumors.

To enhance the cytotoxicity of TPZ to tumor cells, researchers have developed many derivatives of TPZ. Hay *et al.* prepared a series of DNA-targeted TPZ derivatives that increase in cytotoxicity while maintaining hypoxic selectivity [25]. Hicks *et al.* identified that TPZ analogs improved tissue penetration and hypoxic cell killing in tumors [26]. Urea is widely used as the dominant structure in the drug design to establish key drug–target interactions and fine-tune crucial drug-like properties [27–30]. In recent years, FDA has approved many urea-containing compounds for a variety of human diseases, such as Sorafenib [31], Ritonavir [32], Lisuride [33], Lenvatinib [34] and Cariprazine [35]. Developing derivatives of TPZ that contain urea may improve its cytotoxicity to tumor cells.

**Scheme 1.**
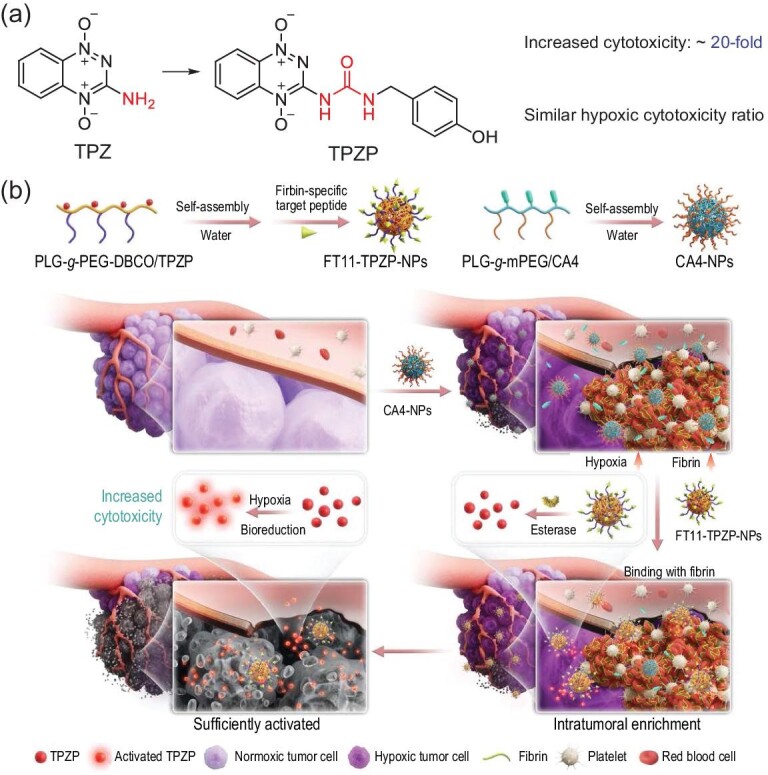
Profile of the tumor targeted drug delivery nanosystem. (a) Chemical structure of TPZP. (b) Schematic diagram of combined therapy.

In this study, we synthesized a series of urea-containing TPZ derivatives (UTPZs), and a derivative (TPZP) with increased hypoxic cytotoxicity (20-fold) and similar hypoxic cytotoxicity ratio (HCR) value compared with TPZ was screened. To ensure that TPZP can effectively enrich and sufficiently activate at the tumor site to exert its antitumor effect, it was prepared as a nanodrug with fibrin-targeting ability. Specifically, TPZP was grafted onto the poly (_L_-glutamic acid)-*graft*-poly(ethylene glycol)-dibenzocyclooctyne (PLG-*g*-PEG-DBCO) to prepare TPZP nanoparticles (TPZP-NPs). Then, FT11, a fibrin-binding peptide, was decorated onto TPZP-NPs using the Copper free Click Chemistry to prepare the FT11-TPZP-NPs. CA4-NPs were introduced to induce coagulation and heavy hypoxia of tumors [21]. As shown in Scheme 1, CA4-NPs precisely destroyed tumor blood vessels, induced tumor cell death and increased the fibrin level (first step); FT11-TPZP-NPs resided at the tumor site by FT11 bonding to fibrin in blood clots (second step); FT11-TPZP-NPs released TPZP in the tumor under the catalysis of esterase (third step); TPZP was effectively activated to kill tumor cells due to CA4-NPs destroying tumor blood vessels and increasing tumor hypoxia (fourth step). In conclusion, this therapeutic strategy led to improved antitumor and anti-metastatic efficacy with marginal side effects.

## RESULTS AND DISCUSSION

### Synthesis and characterization of UTPZs

The synthesis routes of UTPZs are shown in Table 1. In order to conveniently synthesize the UTPZs, we prepared the 3-(((4-nitrophenoxy) carbonyl) amino) benzo[e][1,2,4]triazine 1,4-dioxide (TPZ-NPC) first by reacting TPZ and 4-nitrophenyl chloroformate (NPC). The chemical structure of TPZ-NPC was characterized by ^1^H NMR (Fig. S1) and ^13^C NMR (Fig. S2) spectra. The four peaks in the ^1^H NMR at δ 7.5∼8.5 ppm had the same hydrogen amount belonging to the aryl rings, which showed that we had successfully obtained the intermediate compound TPZ-NPC. Next, we synthesized seven urea-containing derivatives of TPZ with different structures. We characterized these structures of UTPZs through ^1^H NMR and ^13^C NMR, respectively: TPZE (Figs S3 and S4), TPZD (Figs S5 and S6), TPZF (Figs S7 and S8), TPZA (Figs S9 and S10), TPZC (Figs S11 and S12), TPZY (Figs S13 and S14) and TPZP (Figs S15 and S16). All the UTPZs were successfully synthesized through the reactions between TPZ-NPC and the corresponding primary amine compounds.

**Table 1.** Optimization of urea-containing TPZ derivatives (UTPZs).

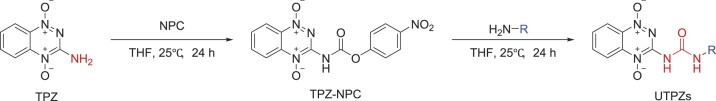					
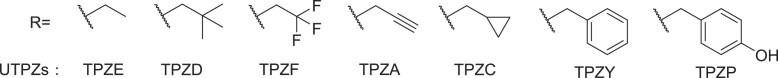					
Compound	Nor: IC_50_ (*µ*m)*^a^*	Hyp: IC_50_ (*µ*m)*^b^*	HCR*^c^*	CTR (Nor)*^d^*	CTR (Hyp)*^e^*
TPZ	51.42	16.35	3.14	–	–
TPZE	4.10	1.16	3.53	12.54	14.09
TPZD	4.33	1.72	2.52	11.88	9.51
TPZF	4.55	1.41	3.23	11.30	11.60
TPZA	3.80	1.13	3.36	13.53	14.47
TPZC	4.30	1.71	2.51	11.96	9.56
TPZY	1.85	0.53	3.49	27.79	30.85
**TPZP**	**2.35**	**0.78**	**3.01**	**21.88**	**20.96**

a Half maximal inhibitory concentration under normoxic conditions. ^b^Half maximal inhibitory concentration under hypoxic conditions. ^c^The hypoxia cytotoxicity ratio. ^d^The cytotoxicity ratio of UTPZs to TPZ under normoxic conditions. ^e^The cytotoxicity ratio of UTPZs to TPZ under hypoxic conditions.

### *In vitro* cytotoxicity assays

To screen a derivative of TPZ with high cytotoxicity while maintaining hypoxic selectivity, CT26 murine colorectal tumor cells were used to evaluate the *in vitro* cytotoxicity of UTPZs through cell viability CCK-8 tests. The CT26 cells were incubated with various concentrations of each UTPZ under normoxic (∼20% O_2_) or hypoxic (∼1% O_2_) conditions for 24 h. As shown in Table 1, the half maximal inhibitory concentration (IC_50_) of TPZ was 51.42 *µ*m under normoxic conditions and 16.35 *µ*m under hypoxic conditions. The HCR of TPZ was 3.14. Interestingly, under normoxic or hypoxic conditions, all the UTPZs were considerably more toxic than TPZ. This suggests that introducing urea can effectively improve the drug efficacy of TPZ. A study has shown that the concept of DNA targeting can effectively enhance the cytotoxicity of TPZ derivatives while maintaining hypoxic selectivity [25]. We speculate that the increased binding affinity with DNA, attributable to the incorporation of urea groups, is the primary factor contributing to the elevated toxicity observed in UTPZs. Especially, the IC_50_ of TPZY was as low as 1.85 *µ*m under normoxic conditions and 0.53 *µ*m under hypoxic conditions. Additionally, the cytotoxicity ratio (CTR) of TPZY to TPZ was ≤27.79 under normoxic conditions and ≤30.85 under hypoxic conditions. This result indicates that introducing the benzene ring can further enhance the cytotoxicity of UTPZs. The IC_50_ of TPZP, which has a reactive phenolic hydroxyl group, was 2.35 *µ*m under normoxic conditions and 0.78 *µ*m under hypoxic conditions. The HCR of TPZP was 3.01, which is similar to that of TPZ (HCR = 3.14). The CTR of TPZP to TPZ was 21.88 under normoxic conditions and 20.96 under hypoxic conditions. The results shown above indicate that TPZP had higher cytotoxicity than TPZ while maintaining hypoxic selectivity. Therefore, TPZP, which is easily chemically modified, was selected for subsequent experiments.

### Synthesis and characterization of FT11-TPZP-NPs

**Figure 1.**
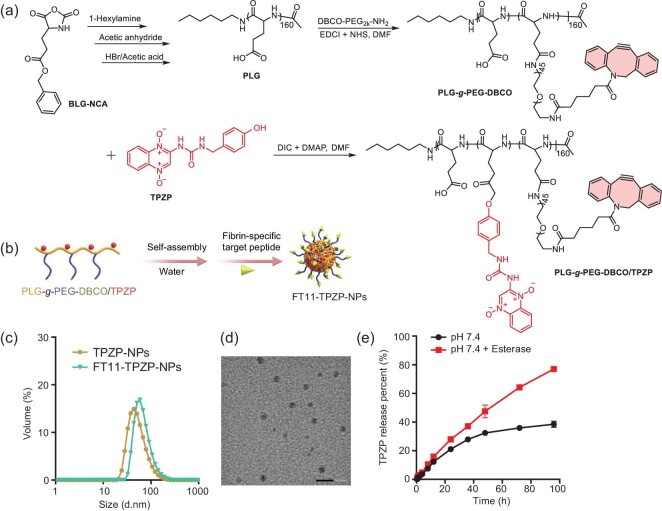
Synthesis and physicochemical characterization of FT11-TPZP-NPs. (a) Synthesis route of PLG-*g*-PEG-DBCO/TPZP. (b) Schematic image of the preparation of FT11-TPZP-NPs. (c) Hydrodynamic size of TPZP-NPs in water for injection (55.0 nm of size and 0.162 of PDI) and FT11-TPZP-NPs (69.6 nm of size and 0.174 of PDI) determined using dynamic light scattering (DLS). (d) TEM image of FT11-TPZP-NPs; scale bar = 100 nm. (e) *In vitro* release of TPZP from FT11-TPZP-NPs in PBS at pH 7.4 with or without esterase.

Nanotechnology plays an important role in the field of medicine and drug delivery, such as improving drug accumulation in tumors and reducing the side effect of drugs to healthy tissues [36,37]. As a biocompatible polymeric backbone, poly(_L_-glutamic acid) (PLG) has entered many clinical trials [38]. To achieve effective enrichment of TPZP in tumors and reduce its side effects, PLG was used to prepared the nanodrug of TPZP. As shown in Fig. 1a, PLG was synthesized through the ring-opening polymerization of *γ*-benzyl-L-glutamate N-carboxyanhydrides (BLG-NCA) and subsequent deprotection [24]. There were no ^1^H NMR signals belonging to the *γ*-benzyl group, indicating that PLG was synthesized (Fig. S17). In order to bond fibrin-targeting peptide that contains the -N_3_ group, we prepared PLG-*g*-PEG-DBCO, which contains DBCO groups, as shown in Fig. 1a. The peak at δ 3.69 ppm (f) belongs to the -CH_2_- in PEG, and the peak at δ 7.25 ppm (g) corresponds to the DBCO group, which showed that the PLG-*g*-PEG-DBCO was successfully synthesized (Fig. S18). Gel permeation chromatography (GPC) analysis indicated that the polydispersity of PLG-*g*-PEG-DBCO was 1.42 and PLG-*g*-PEG-DBCO exhibited a shorter retention time than PLG (Fig. S19). Next, we bonded TPZP to PLG-*g*-PEG-DBCO using an esterification reaction and modified fibrin-targeting peptide (FT11) to achieve effective tumor enrichment. In Fig. S20, the peaks at δ 8.25∼8.49 ppm (g) were attributed to the benzotriazine group, indicating the successful synthesis of PLG-*g*-PEG-DBCO/TPZP conjugates. After the carboxyl groups of PLG were salinized, the conjugates self-assembled to form the TPZP-NPs and were subsequently modified with FT11 peptide using the Copper free Click Chemistry (Fig. 1b). In the ^1^H NMR spectrum of FT11-PLG-*g*-PEG-DBCO/TPZP, we found the characteristic peaks at δ 8.52 ppm (p), δ 4.37 ppm (s) and δ 0.93 ppm (k, l, m, n), which belong to FT11. This showed that we had successfully modified the FT11 peptide to TPZP-NPs for preparing FT11-TPZP-NPs. As shown in Fig. 1c, the hydrodynamic size of TPZP-NPs and FT11-TPZP-NPs was ∼55.0 and ∼69.6 nm, respectively. The polydispersity index (PDI) of the hydrodynamic size of TPZP-NPs and FT11-TPZP-NPs was 0.162 and 0.174, respectively. The FT11-TPZP-NPs exhibited uniformly spherical morphology in transmission electron microscopy (TEM) with a size of ∼36.6 nm (Fig. 1d). These results indicated that both TPZP-NPs and FT11-TPZP-NPs exhibit good hydrodynamic size and distribution. Moreover, the modification of FT11 peptide on the surface of TPZP-NPs did not significantly impact the size. Additionally, FT11-TPZP-NPs remained stable in phosphate buffered saline (PBS) with or without serum at pH 7.4 for 120 h, with no significant change in particle size (Fig. S21). As reported, the ester bond could be effectively and specifically hydrolysed by esterase, which is an overexpressed enzyme in cancer cells [39]. The cumulative release of TPZP from the nanotherapeutic drugs was tested in PBS at pH 7.4 with and without esterase. As shown in Fig. 1e, the TPZP release from FT11-TPZP-NPs was up to ∼77.0% within 96 h in the presence of esterase. In contrast, only 38.5% of TPZP was released from FT11-TPZP-NPs in the absence of esterase. Next, we used PBS at pH 6.8 to simulate an acidic tumor microenvironment and also observed that esterase accelerated TPZP release (Fig. S22). These results indicate that esterases can accelerate the release of TPZP from its nanodrug. Subsequently, we estimated the hypoxia activation ability of the nanodrug of TPZP on CT26 tumor cells. As shown in Fig. S23, after 24 h of co-incubation under hypoxic conditions, both TPZP-NPs and FT11-TPZP-NPs exhibited significantly higher toxicity to CT26 cells compared with normoxic conditions. These results suggest that the nanodrug of TPZP has hypoxic selective toxicity.

### CA4-NPs induced coagulation cascade and heavy hypoxia in tumors

**Figure 2.**
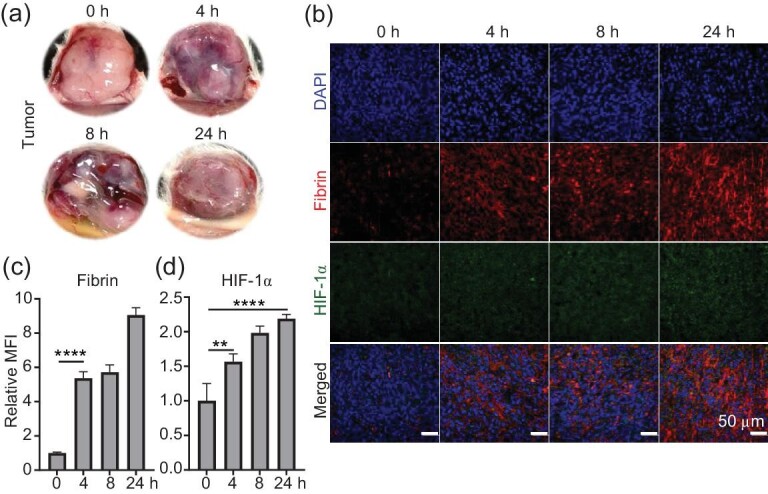
CA4-NPs induced coagulation cascade and heavy hypoxia in tumors. (a) The hemorrhage in the tumors after administration of CA4-NPs over time. (b) Immunofluorescence images of the CT26 tumors after the injection of CA4-NPs (20 mg/kg, eq. to CA4). The tumors were immunostained with anti-fibrin antibody for the fibrin (red) and anti-HIF-1α antibody for the expression of HIF-1α (green). Scale bars, 50 μm. (c) Quantitative data of the fluorescence intensity of fibrin in the immunofluorescence images. (d) Quantitative data of the fluorescence intensity of HIF-1α in the immunofluorescence images. The fibrin and HIF-1α expressions were quantified using fluorescence intensity quantification of the images with Image J. Data are shown as the mean ± SD. Significance between each group was calculated using one-way analysis of variance (ANOVA) with the Tukey post hoc test. **P < 0.01; ****P < 0.0001.

To achieve effective TPZP enrichment and efficient hypoxia activation of FT11-TPZP-NPs in tumors, we introduced CA4-NPs (Fig. S24) into the treatment system for combined treatment. CA4-NPs can induce coagulation and exacerbate hypoxia in the tumors. During the coagulation cascade process, there is an increase in the fibrin level [40–42]. In order to evaluate the hemorrhage situation in mouse tumors after CA4-NPs treatment, we took photos of tumors at different time points. As shown in Fig. 2a, hemorrhage was clearly observed in the tumors at 4 and 8 h after CA4-NPs treatment, which significantly weakened at 24 h. This result indicates that CA4-NPs, as an excellent VDA, can quickly destroy tumor blood vessels. According to literature reports, CA4-NPs display high tumor selectivity, which can cause intratumoral hemorrhage specifically without triggering nonspecific hemorrhage in major organs [21]. To further evaluate the increase in targets and the exacerbation in hypoxia levels, we utilized the immunofluorescence staining technique to investigate the accumulation of fibrin and the expression of hypoxia-inducible factor-1 alpha (HIF-1α) in tumor tissues. According to the images in Fig. 2b, after treatment with CA4-NPs, the aggregation of fibrin in the tumor gradually increased over time. Quantification data also showed that mice treated with CA4-NPs exhibited a significant increase in fibrin within the tumor. The expression of fibrin was ∼5-fold higher at 4 h and ∼9-fold higher at 24 h compared with the control group (0 h). The high expression of fibrin, as a target of FT11 polypeptide, provides the possibility for the effective enrichment of FT11-TPZP-NPs in tumors. The oxygen level must be sufficiently low for TPZP to effectively activate and exert its killing effect on tumor cells. Additionally, the cytotoxicity of TPZP is closely dependent on the degree of hypoxia in the tumor. In order to evaluate the degree of hypoxia in the tumor after CA4-NPs treatment, we assayed the expression level of HIF-1α, a biological marker for exacerbation of hypoxia [43]. As shown in Fig. 2b, after treatment with CA4-NPs, the expression of HIF-1α in tumors increases over time. Further quantification showed that at 4, 8 and 24 h after CA4-NPs treatment, the expression of HIF-1α was 1.6-fold, 2.0-fold and 2.2-fold higher than that of the control group, respectively. Similarly, the hypoxia probe staining assay experiment also demonstrated that CA4-NPs exacerbate tumor hypoxia (Fig. S25). These results indicate that treatment with CA4-NPs can significantly reduce the oxygen content of tumors, increase tumor hypoxia and thus enhance the cytotoxicity of TPZP to tumor cells. In summary, after CA4-NPs treatment, the aggregation of fibrin within the tumor significantly increased, and the degree of hypoxia significantly improved. It is expected to achieve a large accumulation of FT11-TPZP-NPs within the tumor and efficiently activate it, thereby exerting a powerful antitumor effect.

### *In vivo* tumor targeting and toxicity analysis

**Figure 3.**
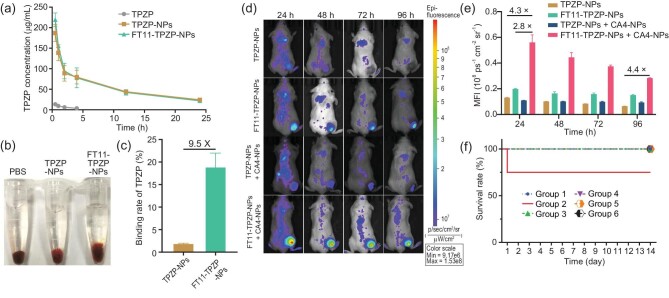
Prolonged blood circulation and coagulation targeting based on long-term presence of the drug in the tumor and reduced side effects of FT11-TPZP-NPs *in vivo*. (a) Plasma pharmacokinetics of TPZP, TPZP-NPs and FT11-TPZP-NPs in Sprague–Dawley rats (*n* = 3). (b) Images of a blood clot in the PBS, TPZP-NPs or FT11-TPZP-NPs. (c) The corresponding TPZP content in the blood clots treated with TPZP-NPs or FT11-TPZP-NPs was evaluated using high-performance liquid chromatography (HPLC) (*n* = 3). (d) Real-time *in vivo* fluorescence imaging of CT26 tumor-bearing BALB/C mice after intravenous injection of Cy7-labeled drugs. (e) The corresponding mean fluorescence intensity value of each tumor collected from (d). (f) The survival rate of mice treated with different dosages of TPZP or TPZP-NPs within 14 days was monitored and recorded (*n* = 4). Groups: (1) TPZP 10 mg/kg; (2) TPZP 20 mg/kg—one in four mice was dead after being treated with TPZP on Day 1; (3) TPZP-NPs, 20 mg/kg, eq. to TPZP; (4) TPZP-NPs, 40 mg/kg, eq. to TPZP; (5) TPZP-NPs, 60 mg/kg, eq. to TPZP; (6) TPZP-NPs, 80 mg/kg, eq. to TPZP.

The plasma pharmacokinetics of the drugs were evaluated on Sprague–Dawley (SD) rats. The half-life (*t*_1/2_) was 2.3, 12.7 and 11.8 h for free TPZP, TPZP-NPs and FT11-TPZP-NPs, respectively (Fig. 3a and Table S1). This result indicates that the nanodrugs of TPZP significantly prolong the blood circulation of the drugs. Additionally, the two nanodrugs had similar blood clearance kinetics. To evaluate the ability of the drug to bind fibrin, we first conducted an *in vitro* clot binding assay. We incubated TPZP-NPs and FT11-TPZP-NPs with freshly obtained mouse blood for 4 h. The clot volume in the FT11-TPZP-NPs group was significantly larger than that in the TPZP-NPs group (Fig. 3b), and the total amount of TPZP in the clots of the FT11-TPZP-NPs group was 9.5-fold higher than that in the TPZP-NPs group (Fig. 3c). These results indicate that FT11-TPZP-NPs have a highly binding efficiency to blood clots. Then, the biodistribution was evaluated at different time points after treatment with TPZP or TPZP-NPs using HPLC tests (Fig. S26). The intratumoral total TPZP concentration in the TPZP-NPs group increased gradually within 24 h. The total TPZP in tumors after TPZP-NPs treatment was 6.5-fold higher after TPZP treatment at 24 h. These results suggest the accumulation of nanoparticles in tumors. Next, to investigate whether the FT11-TPZP-NPs can effectively target the tumors via CA4-NPs-induced coagulation, the mice were imaged in real time at different time points using an *in vivo* fluorescence imaging system (IVIS) after being treated with FT11-TPZP-NPs (labeled with the fluorophore Cy7). As shown in Fig. 3d, at 24 h, the fluorescence intensity of the FT11-TPZP-NPs and FT11-TPZP-NPs + CA4-NPs groups was significantly stronger than that of the TPZP-NPs and TPZP-NPs + CA4-NPs groups, with the FT11-TPZP-NPs + CA4-NPs group having the strongest fluorescence intensity. According to quantitative data, the intratumoral accumulation of the drug in mice treated with FT11-TPZP-NPs + CA4-NPs was ∼2.8-fold higher than that treated with FT11-TPZP-NPs alone and 4.3-fold higher than that treated with TPZP-NPs alone, indicating that the administration of CA4-NPs effectively facilitates the enrichment of FT11-TPZP-NPs in tumors (Fig. 3e). Markedly, by comparing the FT11-TPZP-NPs group with the TPZP-NPs group, it can be concluded that the surface modification of nanoparticles with fibrin-targeting peptides enhanced the ability of the drug to accumulate and remain in tumors. As shown in Fig. 3e, after treatment with TPZP-NPs, the fluorescence intensity of tumors decreased rapidly over time, almost completely disappearing at 72 h. Markedly, after treatment with FT11-TPZP-NPs + CA4-NPs, there was still strong fluorescence inside the tumor, indicating a high concentration of drugs in the tumor even at 96 h. Subsequently, the maximum tolerated doses (MTDs) of TPZP and TPZP-NPs were determined by monitoring the survival of mice after administering different doses of the drug. TPZP-NPs exhibited an excellent safety profile even at a dose of 80 mg/kg (eq. to TPZP); in contrast, TPZP at a dose of 20 mg/kg caused mice to die. The MTDs were confirmed to be 10 mg/kg for TPZP and >80 mg/kg (eq. to TPZP) for TPZP-NPs (Fig. 3f and Fig. S27). These results indicate that the nano formulation of TPZP significantly reduces its systemic toxicity.

### FT11-TPZP-NPs synergized with CA4-NPs effectively inhibited tumor growth

**Figure 4.**
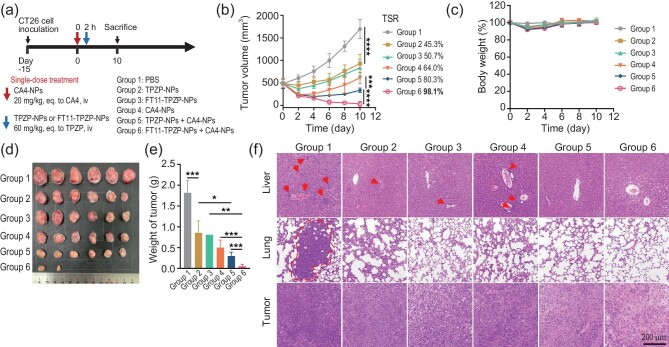
Tumor inhibition of FT11-TPZP-NPs and their combination with CA4-NPs in CT26 tumor model. (a) The treatment schedule; (b) tumor growth curve; (c) body weight changes; (d) tumor morphologies after experiment terminated; (e) the weight of tumors; (f) H&E staining of liver, lung and tumor after different treatments. Scale bar = 200 *µ*m. Groups: (1) PBS; (2) TPZP-NPs; (3) FT11-TPZP-NPs; (4) CA4-NPs; (5) TPZP-NPs + CA4-NPs; (6) FT11-TPZP-NPs + CA4-NPs. *P < 0.05; **P < 0.01; ***P < 0.001; ****P < 0.0001.

Subsequently, the antitumor efficacy was evaluated. As shown in Fig. 4a, the BALB/c mice bearing CT26 tumors with an initial average tumor volume of ∼480 mm^3^ were randomly divided into six groups. Considering the efficient enrichment ability of FT11-TPZP-NPs at the tumor site, we attempted to conduct a single-dose treatment. According to Fig. 4b, on Day 10 post drug administration, as monotherapies, TPZP-NPs, FT11-TPZP-NPs and CA4-NPs had a tumor suppression rate (TSR) of 45.3%, 50.7% and 64.0%, respectively. As expected, combining TPZP-NPs with CA4-NPs resulted in significantly suppressed tumor growth, with a TSR of 80.3%. Notably, combining FT11-TPZP-NPs with CA4-NPs resulted in even more remarkable tumor suppression, with a TSR of 98.1%. On the second day after drug treatment, the weight of the mice slightly decreased, but quickly recovered afterwards (Fig. 4c). After being treated with FT11-TPZP-NPs + CA4-NPs, four out of six tumors in mice had completely regressed and maintained the lowest tumor weight (Fig. 4d and e). To further assess the therapeutic effects, the excised tumors were collected and stained with hematoxylin and eosin (H&E) [37]. As shown in Fig. 4f, the H&E staining of the liver and lung showed a large area of metastatic nodules in the PBS group, and many areas with small metastatic nodules in the liver were observed in the monotherapy group. In contrast, the anti-metastatic effect of combination therapy was observed, as no obvious metastatic nodules were found in either the lungs or the liver. It was also found that FT11-TPZP-NPs + CA4-NPs led to extensive death of tumor cells, with no apparent damage to other organs observed (Fig. 4f and Fig. S28). Furthermore, blood chemistry analysis revealed no significant changes in alanine aminotransferase (ALT), aspartate aminotransferase (AST), blood urea nitrogen and creatinine (CRE) levels (Fig. S29). This suggests that treatment with the three drugs did not have any adverse effects on liver and kidney function. The results suggest that increasing the hypoxia of tumors while prolonging the residence of FT11-TPZP-NPs at tumor sites can significantly enhance the therapeutic effect of drugs on tumors, without causing any obvious toxic side effects.

## CONCLUSION

In this study, we proposed to develop TPZ derivatives with high hypoxia cytotoxicity and tumor enrichment capacity to fully exert its anticancer therapy effect. We first found that the urea-containing derivatives of TPZ have higher cytotoxicity than TPZ, while maintaining similar HCR. TPZP was synthesized to a nanodrug with fibrin-targeting ability, namely FT11-TPZP-NPs. By combining treatment with CA4-NPs, FT11-TPZP-NPs achieved highly efficient enrichment and effective activation at tumor sites. Single-dose combination therapy achieved a TSR of ≤98.1% and clearance of four out of six tumors with an initial volume of ∼480 mm^3^. Additionally, the combination significantly inhibited the tumor metastasis. This study provides a new strategy to improve the therapeutic effect of TPZ and HAPs.

## Supplementary Material

nwae038_Supplemental_File
