# Supplementary material for: Introducing urea into tirapazamine derivatives to enhance anticancer therapy
Source: Natl Sci Rev. 2024 Feb 5;11(4):nwae038. doi: 10.1093/nsr/nwae038 (PMC10911816; doi:10.1093/nsr/nwae038)
Supplement: nwae038_Supplemental_File [file nwae038_supplemental_file.pdf]

Supporting Information

Introducing urea into the tirapazamine derivatives to enhance anti-cancer therapy

Yajun Xu[1], Jianlin Lv[1,2], Chaoying Kong[1,2], Ya Liu[1,2], Kun Wang[1], Zhaohui Tang[1,2*], Xuesi Chen[1,2]

[1]Key Laboratory of Polymer Ecomaterials, Changchun Institute of Applied Chemistry, Chinese Academy of Sciences, Changchun 130022, China

[2]School of Applied Chemistry and Engineering, University of Sciences and Technology of China, Hefei 230026, China

*Corresponding authors: ztang@ciac.ac.cn

Experimental Methods

Materials

All reagents and solvents were commercially available and used as received unless otherwise specified purification. Extra dry N, N-dimethylformamide (DMF), extra dry tetrahydrofuran (THF) and 4-Nitrophenyl chloroformate were purchased from Beijing J&K Co., LTD. Tirapazamine (TPZ) was purchased from Biochempartner. DBCO-PEG$_{2k}$-NH$_2$ was purchased from Shanxi New Research Biosciences Co., LTD. Cyanine 7 azide (Cy7-N3) was purchased from Lumiprobe Corporation (Broward, FL, USA). BLG-NCA was purchased from Chengdu Enlai Biological Technology CO., LTD. CA4-NPs was prepared as described in previous studies and its drug loading content was 15.8 wt%[1]. Other commercially available chemicals were purchased from Aladdin-Reagent Co. Ltd. CCK-8, Fetal bovine serum (FBS) and RPMI 1640 Media were purchased from meilunbio Co. Ltd. Esterase from porcine liver was purchased from Merck Ltd. Fibrin-specific targeted (FT11) polypeptide (3-Azidopropanoic Acid-FHC[Hyp][Y(3-Cl)]GLCYIQ-NH$_2$, Y(3-Cl) = 3-chlorotyrosine, Hyp = 4-hydroxyproline) was purchased from ChinaPeptides Co.,Ltd., China.

Characterization

^{1}H NMR spectra and ^{13}C NMR spectra were characterized by Bruker AV-500. Transmission electron microscopy (TEM) imaging was performed on a JEOL JEM-1400 (Tokyo, Japan) with a field emission gun operating at 120 kV. Gel permeation chromatography (GPC) measurement was performed on a Waters 515 GPC system. The Z-Average size of nanoparticles was measured by dynamic light scattering (DLS), which was performed on Malvern Zetasizer instrument (Nano-ZS), with a 173 ℃ backscatter. High-performance liquid chromatography (HPLC) was equipped with a reverse-phase C18 (SinoPak BEH AQ-C18 5 μm) analytical column with a UV-Vis detector; a mobile phase consisting of methanol and water was used at a flow rate of 1.0 mL min^{-1}. The absorption wavelength was set at 266 nm. Histological alterations were observed by optical microscope (Nikon Eclipse Ti, Optical Apparatus Co., Ardmore, PA, USA). Immunofluorescence slides were pictured through a confocal laser scanning microscope (CLSM, Carl Zeiss LSM 700, Germany).

Synthesis of TPZ-NPC

TPZ-NPC was prepared as described in previous studies[2]. TPZ (700 mg, 3.9 mmol) was dissolved in 50.0 mL extra dry THF, then after cooling the solution under an ice-water bath for 5 mins, 4-Nitrophenyl chloroformate (1188.3 mg, 5.9 mmol) in 10 mL extra dry THF was slowly added by syringe, while the temperature was controlled to 25 ℃. After stirring for 24 h, the reaction mixture was concentrated in vacuo. The residue was chromatographed on a silica gel column with ethyl acetate and n-hexane as an eluent to yield compound 3-(((4-nitrophenoxy) carbonyl) amino) benzo[e][1,2,4]triazine 1,4-dioxide (TPZ-NPC) as a yellow powder. The structure was determined by ^{1}H NMR (Figure S1) and ^{13}C NMR (Figure S2) using DMSO-d_6 as the solvent. ^{1}H NMR (500 MHz, DMSO) δ = 8.43 (dd, J=8.7, 1.2, 1H), 8.21 (ddd, J=8.4, 7.3, 1.2, 1H), 8.01 (dd, J=8.4, 1.2, 1H), 7.76 (ddd, J=8.6, 7.3, 1.2, 1H). ^{13}C NMR (126 MHz, DMSO) δ = 159.54, 158.07, 139.01, 132.83, 129.50, 128.06, 122.28, 113.22.

General Procedure for preparation of urea-containing tirapazamine derivatives (UTPZs)

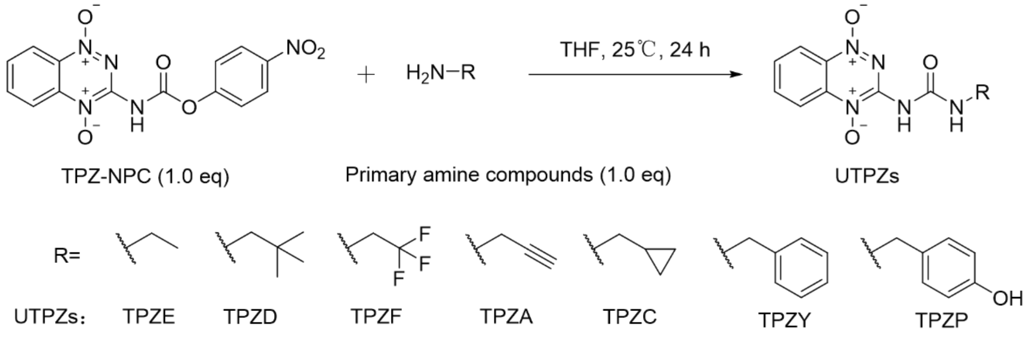

TPZ-NPC (100 mg, 0.3 mmol) was dissolved in 20.0 mL extra dry THF, and the primary amine compound (0.3 mmol) in 10.0 mL extra dry THF was slowly added by syringe, while the temperature was controlled to 25 ℃. After stirring for 24 h, the reaction mixture was concentrated in vacuo. The residue was chromatographed on a silica gel column with methanol and dichloromethane as an eluent to yield UTPZs. The structures of UTPZs were determined by ^{1}H NMR and ^{13}C NMR using DMSO-d_6 as the solvent. TPZE: ^{1}H NMR (500 MHz, DMSO) δ = 9.87 (s, 1H), 8.33 – 8.24 (m, 2H), 8.04 (ddd, J=8.5, 7.0, 1.3, 1H), 7.76 (ddd, J=8.5, 6.9, 1.3, 1H), 7.61 (t, J=5.4, 1H), 3.18 (dddd, J=7.2, 5.4, 2H), 1.10 (t, J=7.2, 3H) (Figure S3); ^{13}C NMR (126 MHz, DMSO) δ = 150.15, 145.80, 137.73, 135.41, 131.80, 129.01, 120.78, 117.66, 54.59, 33.97, 14.57 (Figure S4). TPZD: ^{1}H NMR (500 MHz, DMSO) δ = 10.06 (s, 1H), 8.27 (dddd, J=15.6, 8.7, 1.3, 0.6, 2H), 8.03 (ddd, J=8.6, 7.0, 1.3, 1H), 7.76 (ddd, J=8.7, 7.0, 1.3, 1H), 7.61 (t, J=6.0, 1H), 2.98 (d, J=5.9, 2H), 0.90 (s, 9H) (Figure S5); ^{13}C NMR (126 MHz, DMSO) δ = 150.84, 146.21, 138.06, 135.72, 132.07, 129.32, 121.09,

117.97, 50.67, 31.79, 27.04 (Figure S6). TPZF: ^{1}H NMR (500 MHz, DMSO) δ = 10.26 (s, 1H), 8.30 (ddd, J=14.8, 8.8, 1.2, 2H), 8.06 (ddd, J=8.5, 7.0, 1.3, 1H), 7.79 (ddd, J=8.5, 7.0, 1.3, 1H), 4.04 (qd, J=9.7, 6.3, 2H) (Figure S7); ^{13}C NMR (126 MHz, DMSO) δ = 151.33, 146.31, 138.58, 136.22, 132.94, 130.14, 121.58, 118.56, 40.95 (Figure S8). TPZA: ^{1}H NMR (500 MHz, DMSO) δ = 10.03 (s, 1H), 8.35 – 8.23 (m, 2H), 8.05 (ddd, J=8.5, 7.0, 1.3, 1H), 7.93 (t, J=5.5, 1H), 7.78 (ddd, J=8.5, 7.0, 1.3, 1H), 3.98 (dd, J=5.5, 2.5, 2H), 3.20 (t, J=2.5, 1H) (Figure S9); ^{13}C NMR (126 MHz, DMSO) δ = 150.48, 145.98, 138.11, 135.78, 132.32, 129.55, 121.13, 118.06, 80.94, 73.54, 28.98 (Figure S10). TPZC: ^{1}H NMR (500 MHz, DMSO) δ = 9.95 (s, 1H), 8.29 (dddd, J=16.4, 8.7, 1.3, 0.6, 2H), 8.04 (ddd, J=8.5, 7.0, 1.3, 1H), 7.89 – 7.60 (m, 2H), 3.05 (dd, J=6.9, 5.5, 2H), 1.03 – 0.90 (m, 1H), 0.52 – 0.39 (m, 2H), 0.28 – 0.13 (m, 2H) (Figure S11); ^{13}C NMR (126 MHz, DMSO) δ = 150.92, 146.48, 138.41, 136.06, 132.48, 129.69, 121.45, 118.34, 44.07, 11.20, 3.46 (Figure S12). TPZY: ^{1}H NMR (500 MHz, DMSO) δ = 10.06 (s, 1H), 8.29 (ddd, J=16.8, 8.8, 1.2, 2H), 8.11 – 8.01 (m, 2H), 7.77 (ddd, J=8.5, 7.0, 1.3, 1H), 7.39 – 7.32 (m, 4H), 7.27 (ddt, J=8.6, 5.9, 2.0, 1H), 4.38 (d, J=5.8, 2H) (Figure S13); ^{13}C NMR (126 MHz, DMSO) δ = 151.26, 146.58, 138.57, 136.20, 132.61, 129.86, 121.58, 118.48, 60.31, 42.64 (Figure S14). TPZP: ^{1}H NMR (500 MHz, DMSO) δ = 9.99 (s, 1H), 9.34 (s, 1H), 8.33 – 8.24 (m, 2H), 8.04 (ddd, J=8.5, 7.0, 1.3, 1H), 7.95 (t, J=5.7, 1H), 7.76 (ddd, J=8.5, 7.0, 1.3, 1H), 7.17 – 7.10 (m, 2H), 6.76 – 6.70 (m, 2H), 4.25 (d, J=5.6, 2H) (Figure S15); ^{13}C NMR (126 MHz, DMSO) δ = 156.47, 150.66, 146.15, 138.08, 135.74, 132.18, 129.39, 129.18, 128.72, 121.12, 118.01, 115.14, 42.63 (Figure S16).

Synthesis of poly($_L$-glutamic acid) (PLG)

Poly($_L$-glutamic acid) (PLG) has an average of 160 $_L$-glutamic acid repeating units was prepared as described as previously reported[3]. After recrystallization with extra dry THF and extra dry n-hexane, BLG-NCA (30.0 g, 11.4 mmol) was dissolved in 300 mL extra dry DMF under an argon atmosphere. Then *n*-hexylamine (7.2 mg, 0.071 mmol) in 10 mL extra dry DMF was added slowly to react for another three days at 35 ℃. End of the third day, the triethylamine (72.0 mg, 0.71 mmol) and acetic anhydride (72.6 mg, 0.71 mmol) were added to further react for 24 h at 35 ℃. The reaction mixtures were then precipitated into excess diethyl ether. The formed precipitate was dried at room temperature under reduced pressure, then the formed precipitate was re-dissolved in DMF, and dialyzed against distilled water (MWCO 3500) and PLG was obtained after freeze-drying.

Synthesis of PLG-*g*-PEG-DBCO

PLG (258.0 mg, 2.0 mmol Glu monomer) and DBCO-PEG$_{2k}$-NH$_2$ (100.0 mg, 0.05 mmol) and NHS (6.9 mg, 0.06 mmol) were dissolved in 20 mL extra dry DMF, then EDCI (11.5 mg, 0.06 mmol) and DIPEA (16.2 mg, 0.125 mmol) were added into the reaction mixture. After reacting for 72 h at 25 ℃, the solution was precipitated using an excess amount of diethyl ether and washed twice with diethyl ether. At last, the precipitate was dialyzed against distilled water for 72 h. The PLG-*g*-PEG-DBCO was obtained after freeze-drying.

Synthesis of PLG-TPZP

PLG-TPZP was prepared by Steglich esterification between PLG-*g*-PEG-DBCO and TPZP. Briefly, PLG-*g*-PEG-DBCO (340.0 mg) and TPZP (60.0 mg, 0.184 mmol) were dissolved in 20 mL extra dry DMF, then DIC (32.5 mg, 0.258 mmol) and DMAP (35.8 mg, 0.294 mmol) in 5 mL extra dry DMF were added into the reaction mixture. After reacting for 72 h at 25 ℃, the solution was precipitated using an excess amount of diethyl ether. The formed precipitate was dried at room temperature under reduced pressure, then the formed precipitate was re-dissolved in DMF, and dialyzed against distilled water and PLG-TPZP was obtained after freeze-drying.

Synthesis of FT11-TPZP-NPs

FT11-TPZP-NPs was obtained by the Copper free Click Chemistry based on the reaction of DBCO with azide-labeled polypeptide. Briefly, PLG-TPZP (150.0 mg) was dissolved in 10.0 mL 0.01 M PBS (pH = 7.4) and fibrin-specific targeted peptide (FT11) (31.2 mg) in 2 mL 0.01 M PBS (pH = 7.4) was added to react for 12 h at 37 ℃. After dialyzed against distilled water for 36 h, the FT11-TPZP-NPs was obtained.

***In vitro* drug release under esterase catalysis**

The in *vitro* release of TPZP from FT11-TPZP-NPs was evaluated in 0.01 M PBS (pH = 6.8 or pH = 7.4). Briefly, FT11-TPZP-NPs (12 mg/mL,) was mixed with the same volume aqueous esterase solution (0.4 mg/mL) under a gently shaking rate of 90 rpm at 37 ℃. As a control experiment, FT11-TPZP-NPs (12 mg/mL) was mixed with the same volume 0.01 M PBS. 200 uL mixtures were collected at specific times and diluted with 400 uL methanol for HPLC analysis at 266 nm with a mobile phase of methanol and water (50/50, v/v).

Clot assay *in vitro*

TPZP-NPs or FT11-TPZP-NPs was dissolved in water with a TPZP concentration of 0.2 mg/mL. 0.5 mL of the above solution was collected and incubated with 0.2 mL freshly withdrawn mouse blood at 25 ℃ for 4 h. The fibrin clots were washed with PBS several times and photographed. Then, the fibrin clots were homogenized, hydrolyzed with 1 M NaOH solution for 12 h, neutralized with 1.4 M H_3PO_4. After extracted with methanol, the total amount of TPZP in these fibrin clots was analyzed by HPLC at 266 nm with a mobile phase of methanol and water (50/50, v/v).

Cytotoxicity assay

The CT26 cells were cultured in 96-well plates at a density of 5000 cells per well in 190 μL RPMI 1640 Media overnight. Then, the 10 uL solution containing UTPZs or TPZP-NPs or TPZP-NPs was added. After being cultured in hypoxic or normoxic conditions for 24 h, 20 μL CCK-8 solution was added, and the samples were measured using a TECAN Infinite F50 at 450 nm[4]. For hypoxic conditions, the cells received a continuous flow of a humidified mixture of 94% N_2, 5% CO_2, and 1% O_2 for 5 mins, then incubated at 37 ℃.

Maximum tolerance dose (MTD) studies

The MTD studies were carried out on Female BALB/C mice. The mice were randomly divided into ten groups (n = 4) and injected with TPZP (at a dose of 10, 20 mg/kg) or TPZP-NPs (at a dose of 20, 40, 60, 80 mg/kg, eq. to TPZP) via tail vein. After one injection, the body weight of the mice was recorded every day, and whether the mice died or not was observed. The MTD was defined as the dose of the drug that did not cause the animal to die during the experiment.

Plasma pharmacokinetics

SD rats (n = 3, female, average body weight 200 g) were treated with TPZP-NPs and TPZP through an intravenous injection in the tail vein at a dose of TPZP of 15.0 mg/kg. Blood samples were collected from the orbital cavity at specific time points (0.5, 1, 2, 4, 8, 12 and 24 h), and centrifuged to obtain the plasma. For each sample, 100 μL plasma were hydrolyzed with 1 M NaOH solution for 12 h, neutralized with 1.4 M H_3PO_4, then were extracted with 0.4 mL methanol. The mixture was vortexed and filtered for further measurement using an HPLC system. with a UV-Vis detector at 266 nm to detect TPZP eluted with methanol and water (v/v=50:50).

Fibrin and HIF-1α immunofluorescence

CT26 tumor-bearing mice with the tumor volume reached approximately 480 mm^3 were administrated CA4-NPs (20 mg/kg, eq to CA4) via tail vein. The mice were excised at predetermined time points (4, 8 and 24 h) and mice without drug administration as zero hour. After taking pictures, the tumors of mice were immersed in 4% paraformaldehyde solution for fibrin and HIF-1α immunofluorescence.

Drug distribution *in vivo*

TPZP and TPZP-NPs were intravenously injected into the BALB/c mice bearing CT26 tumors at the TPZP dose of 15 mg/kg. The mice were excised at predetermined time points (4 and 24 h) after the injection. The tumor and main organs were collected and ground. In order to detect the free amount of TPZP from the solution of the ground tissues, the free TPZP was directly extracted with methanol. For detected the total TPZP after injected TPZP-NPs, before extracting with methanol, samples were hydrolyzed with 1 M NaOH solution for 12 h, neutralized with 1.4 M H_3PO_4 to release of TPZP from TPZP-NPs. And then the samples were qualitatively analyzed by HPLC with a mobile phase of methanol and water (50/50, v/v). Cy7-N_3 was used to label TPZP-NPs and FT11-TPZP-NPs for the *in vivo* imaging. After the tumor volume reached approximately 480 mm^3, the mice were randomly divided into four groups and intravenously injected with Cy7-labeled-TPZP-NPs, Cy7-labeled-FT11-TPZP-NPs, CA4-NPs + Cy7-labeled-TPZP-NPs or CA4-NPs + Cy7-labeled-FT11-TPZP-NPs. Cy7-labeled-TPZP-NPs or Cy7-labeled-FT11-TPZP-NPs was injected two hours after the administration of CA4-NPs. The dosage for Cy7-labeled-TPZP-NPs or Cy7-labeled-FT11-TPZP-NPs was 10 mg/kg (eq. to TPZP). The dosage for CA4-NPs was 20 mg/kg (eq. to CA4). At predetermined time points (24, 48, 72 and 96 h), fluorescence *in vivo* was observed by the *in vivo* optical imaging system (IVIS Lumina LT).

***In vivo* antitumor efficacy**

The female BALB/c mice (6–8 weeks old) were firstly subcutaneously embedded with CT26 cells (1×10^6) into the abdomen, and once the tumor volume reached approximately 480 mm^3, the mice were a randomly divide into six groups: 1) PBS; 2) TPZP-NPs; 3) FT11-TPZP-NPs; 4) CA4-NPs; 5) CA4-NPs + TPZP-NPs; 6) CA4-NPs + FT11-TPZP-NPs. TPZP-NPs or FT11-TPZP-NPs was injected intravenously at a dose of 60 mg/kg (eq. to TPZP), and CA4-NPs were injected intravenously at a dose of 20 mg/kg (eq. to CA4). All medicines were given only once on day zero. The tumor sizes and body weights were recorded every two days to monitor antitumor efficiency and safety. On the day 10, the mice were sacrificed. Blood was collected

for complete blood count test and liver and kidney function assessment. After taking pictures, the tumors and main organs (hearts, livers, spleens, lungs, and kidneys) of mice were immersed in 4% paraformaldehyde solution for H&E staining[5]. The tumor volumes were measured with vernier caliper and calculated using the following formula: tumor volume (V) = $1/2 \times a \times b^2$, where a and b represent the major axis and the minor axis of the tumor, respectively. The tumor suppression rates (TSR) were calculated using the formula: TSR (%) = $[(Vc - Vt)/Vc] \times 100\%$, where the Vt and Vc represent the mean tumor volume of the treatment and PBS groups, respectively.

Statistical Analysis

All results were performed as the mean ± standard deviation (s.d.). Data were analyzed by one way analysis of variance (ANOVA) for comparison of multiple groups and unpaired t tests for comparison between two groups. P values less than 0.05 were considered statistically significant.

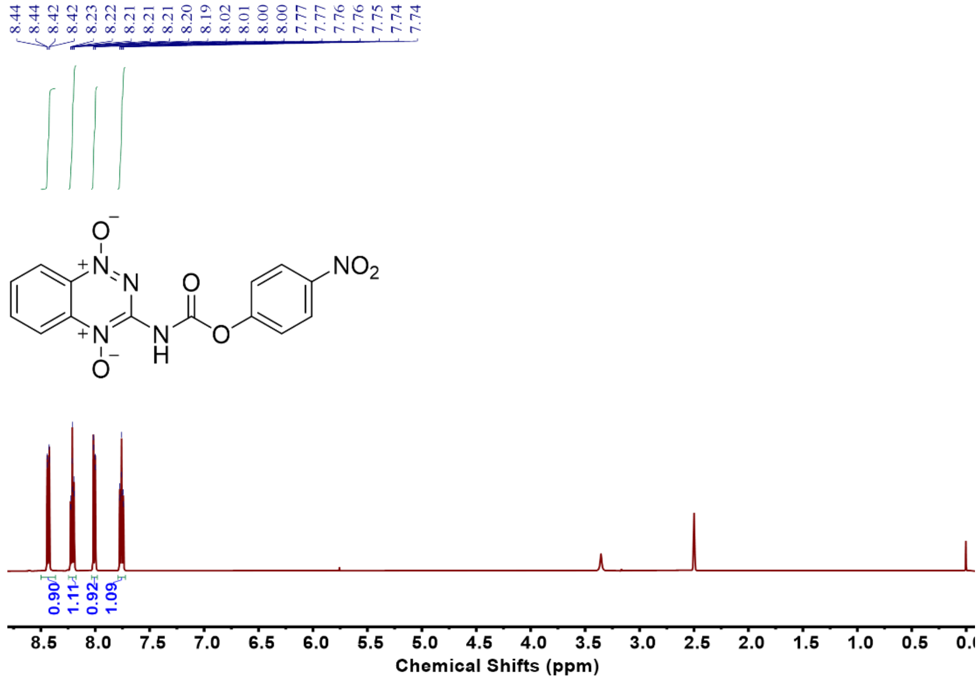

Figure S1. The ^{1}H NMR spectrum of TPZ-NPC.

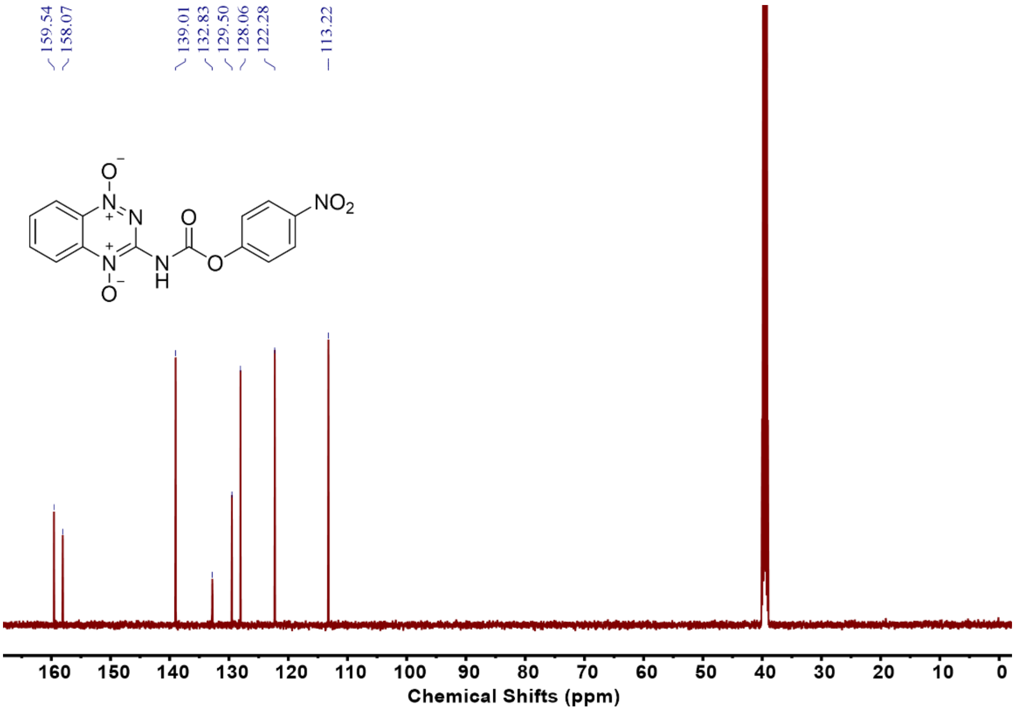

Figure S2. The ^{13}C NMR spectrum of TPZ-NPC.

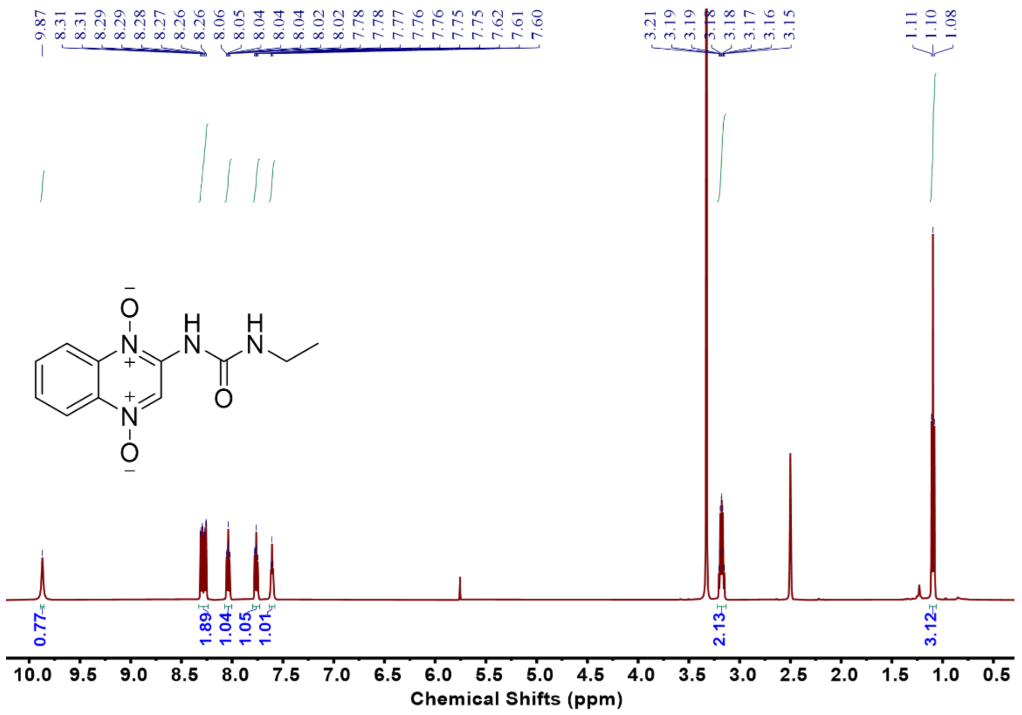

Figure S3. The ^{1}H NMR spectrum of TPZE.

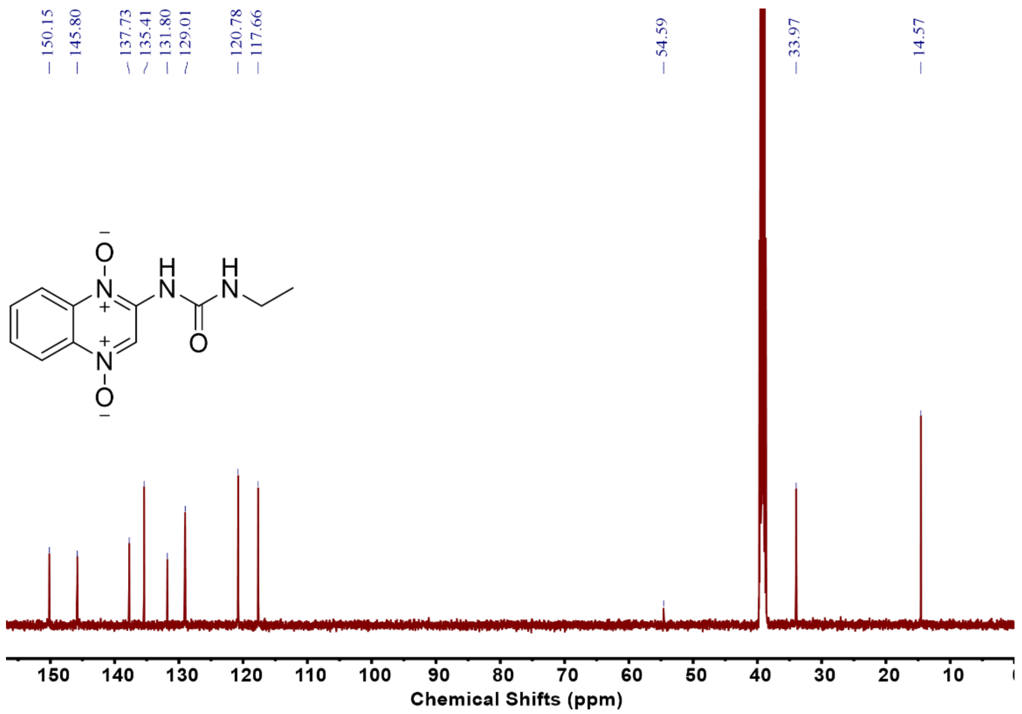

Figure S4. The ^{13}C NMR spectrum of TPZE.

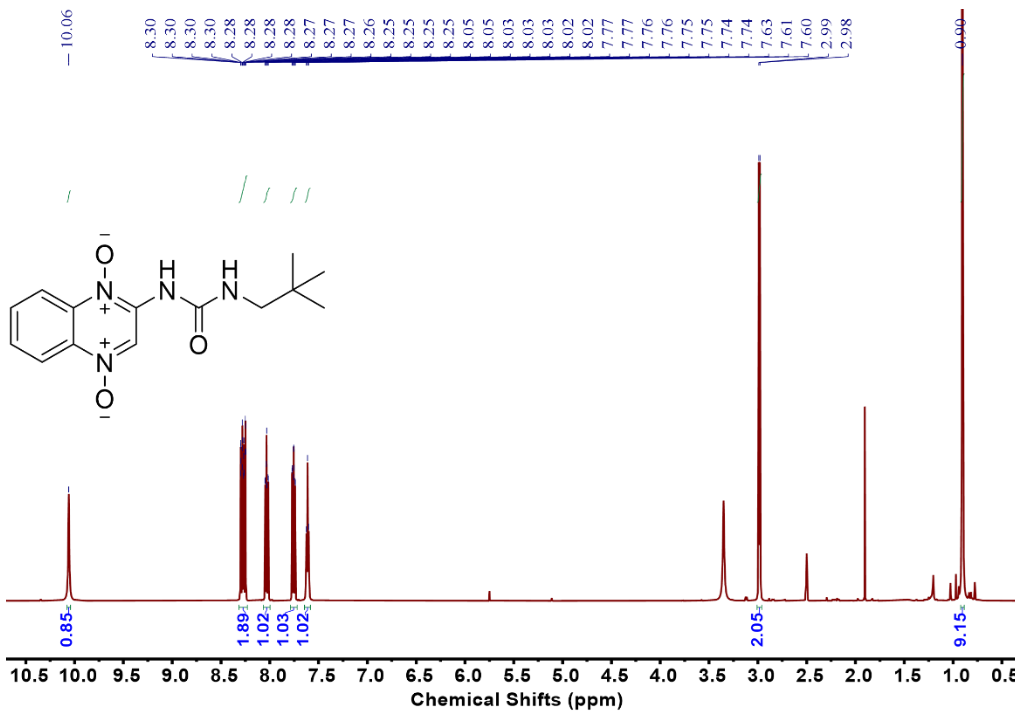

Figure S5. The ^{1}H NMR spectrum of TPZD.

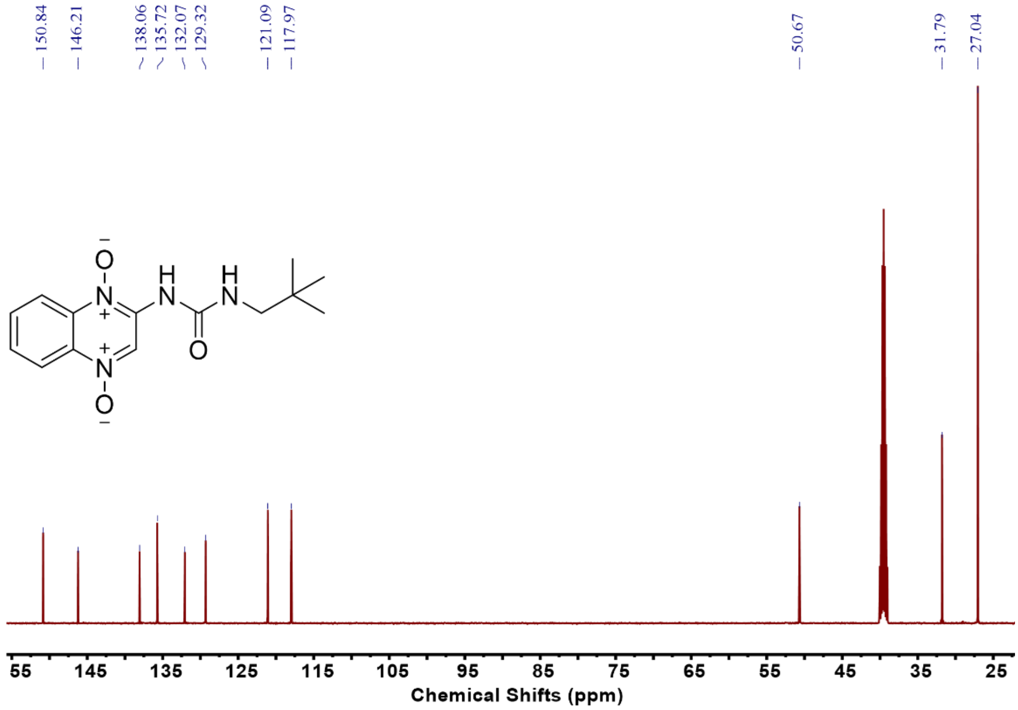

Figure S6. The ^{13}C NMR spectrum of TPZD.

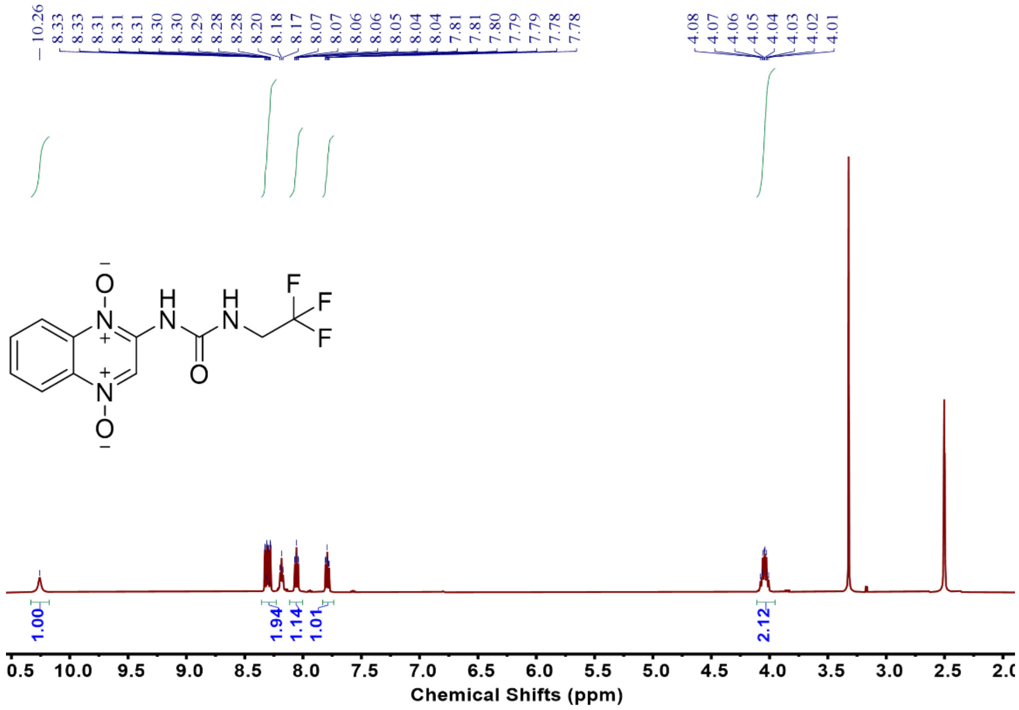

Figure S7. The ^{1}H NMR spectrum of TPZF.

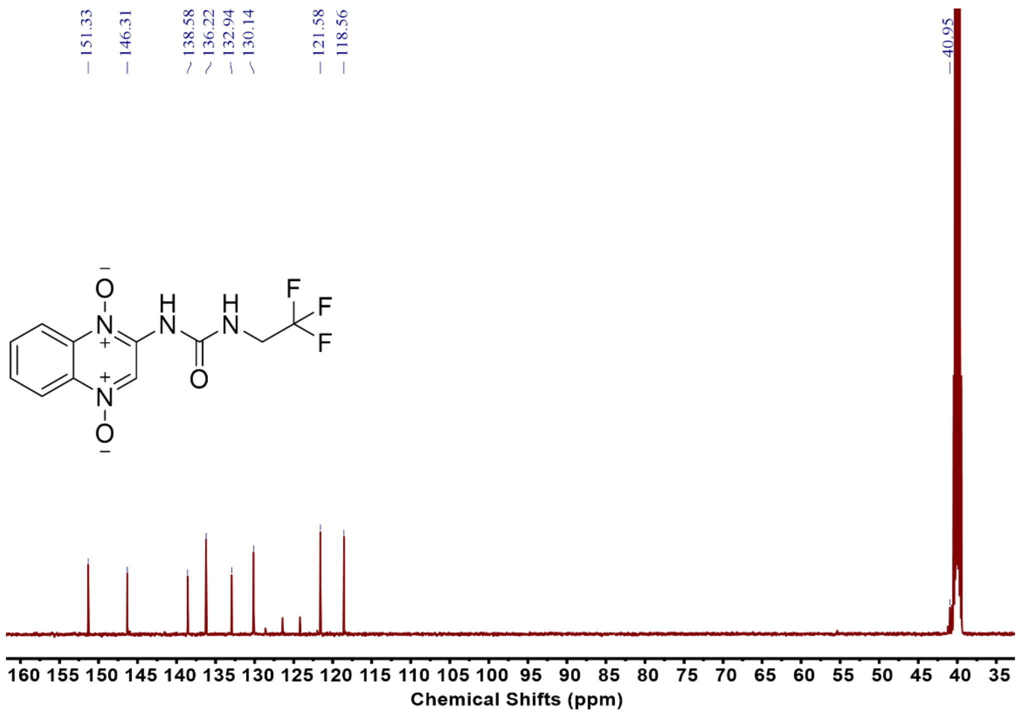

Figure S8. The ^{13}C NMR spectrum of TPZF.

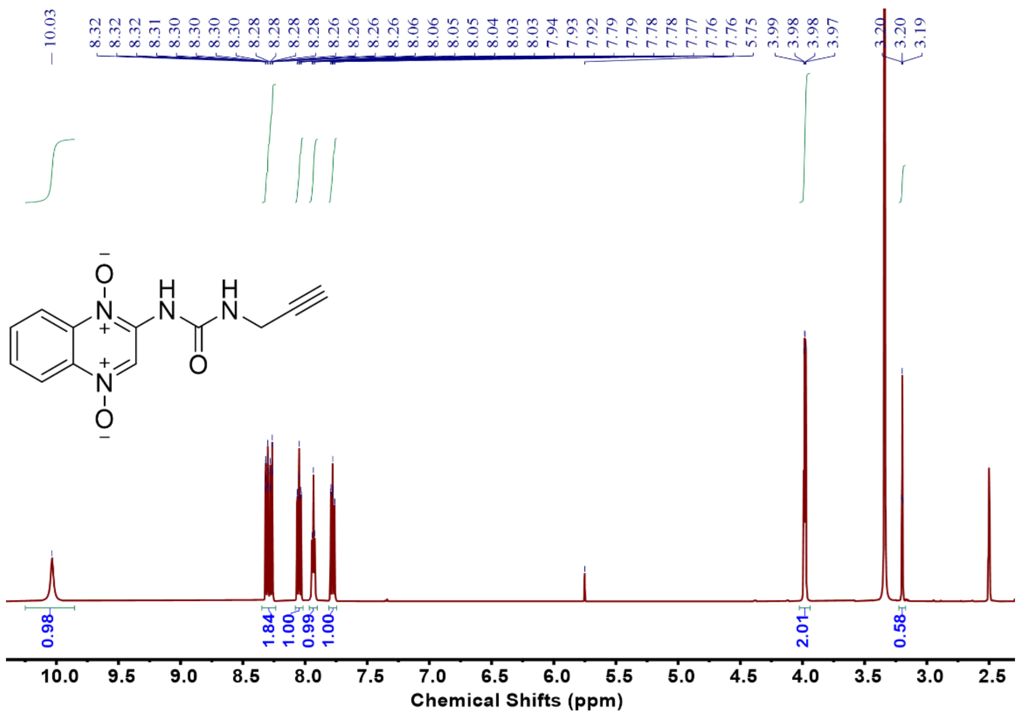

Figure S9. The ^{1}H NMR spectrum of TPZA.

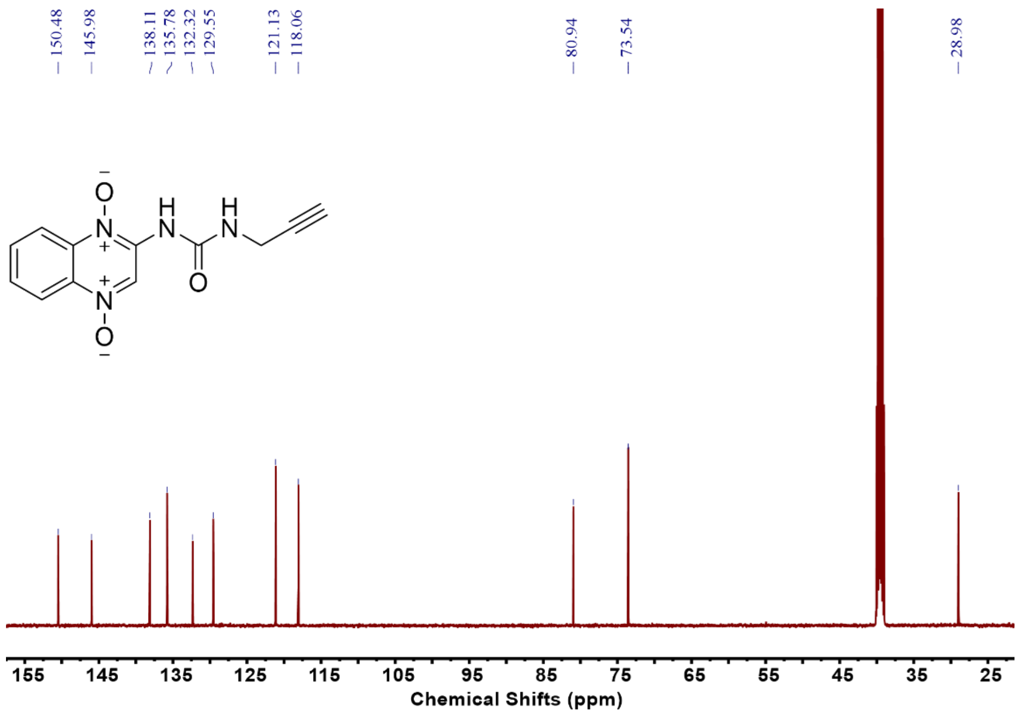

Figure S10. The ^{13}C NMR spectrum of TPZA.

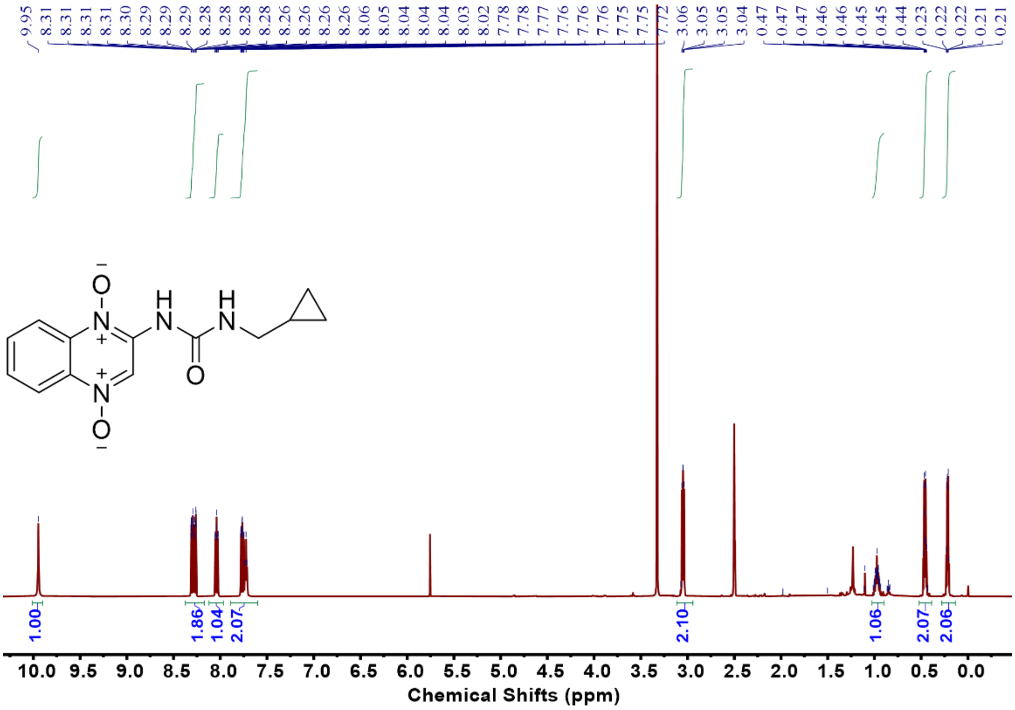

Figure S11. The ^{1}H NMR spectrum of TPZC.

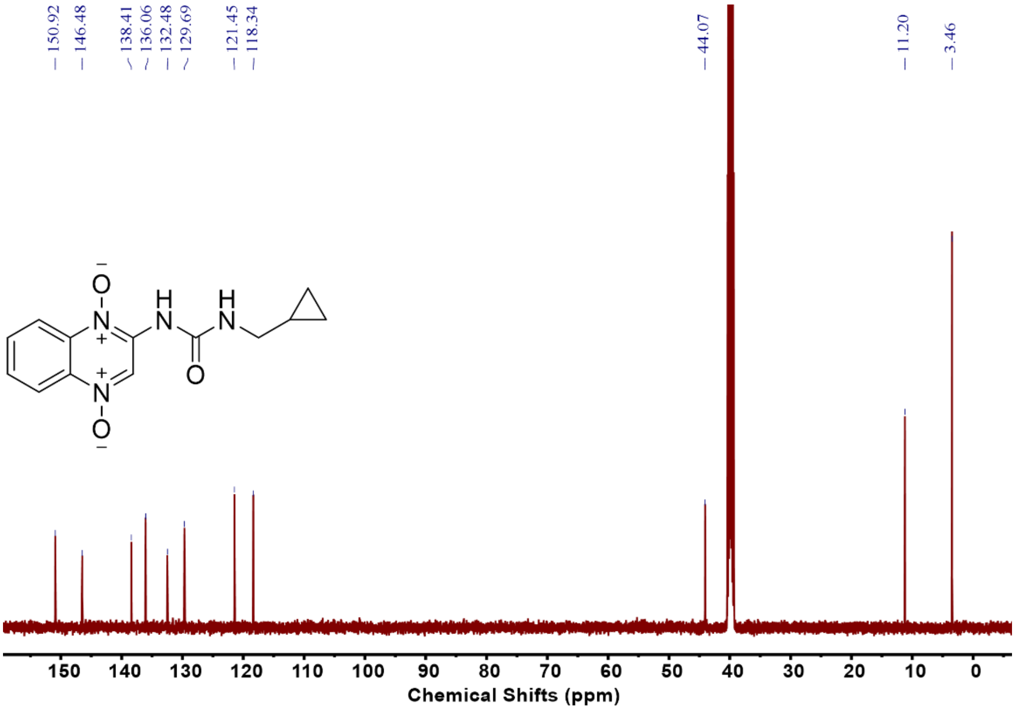

Figure S12. The ^{13}C NMR spectrum of TPZC.

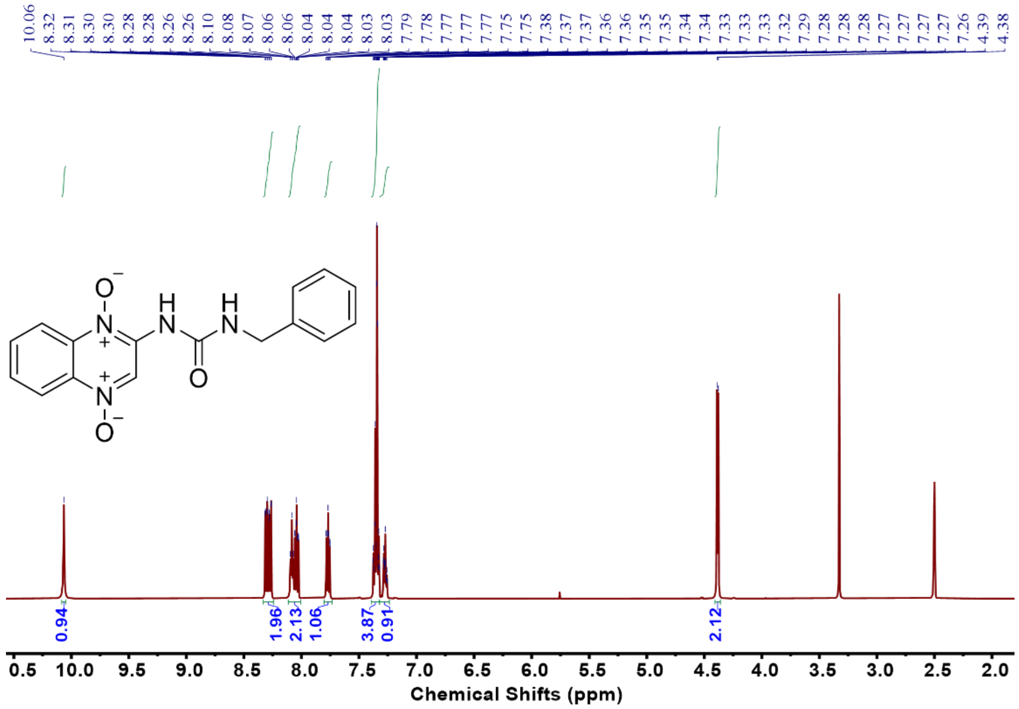

Figure S13. The ^{1}H NMR spectrum of TPZY.

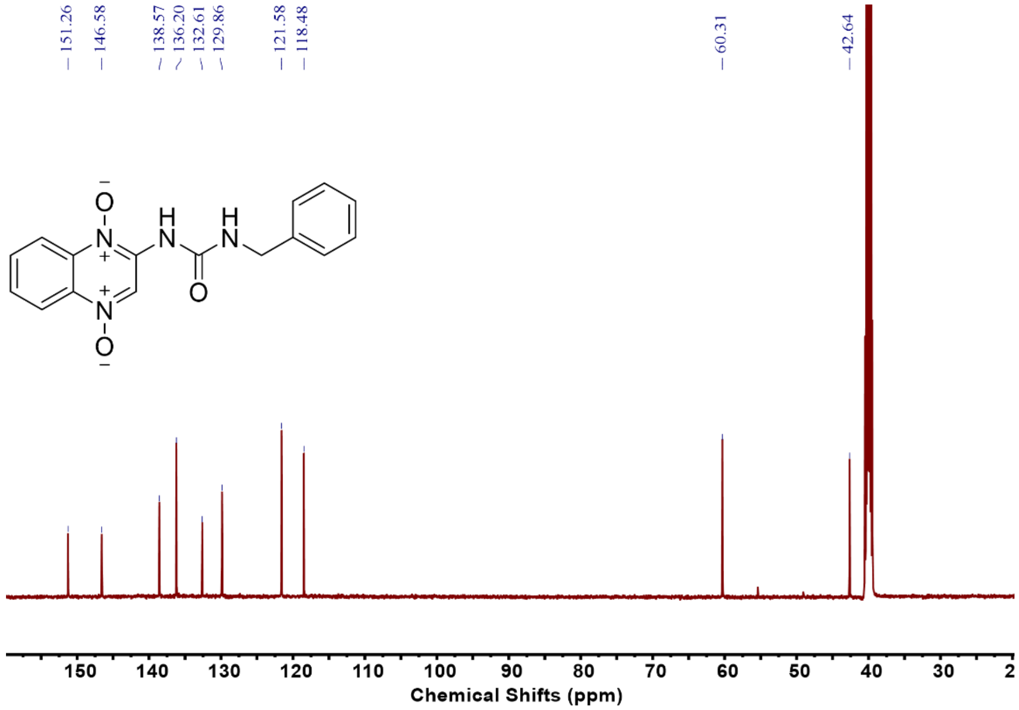

Figure S14. The ^{13}C NMR spectrum of TPZY.

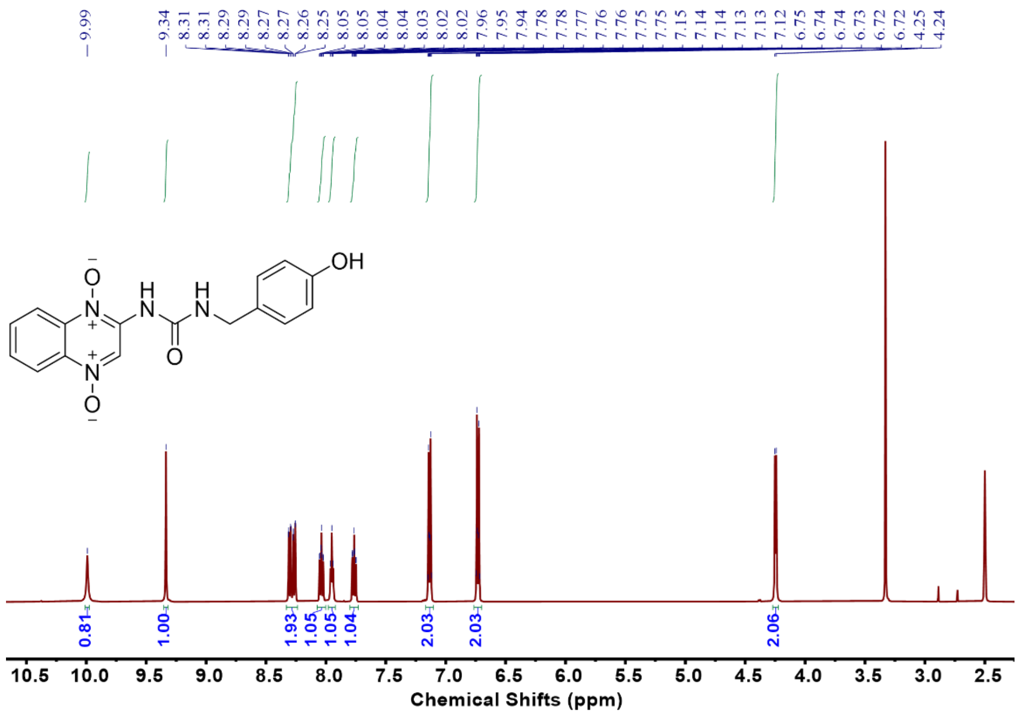

Figure S15. The ^{1}H NMR spectrum of TPZP.

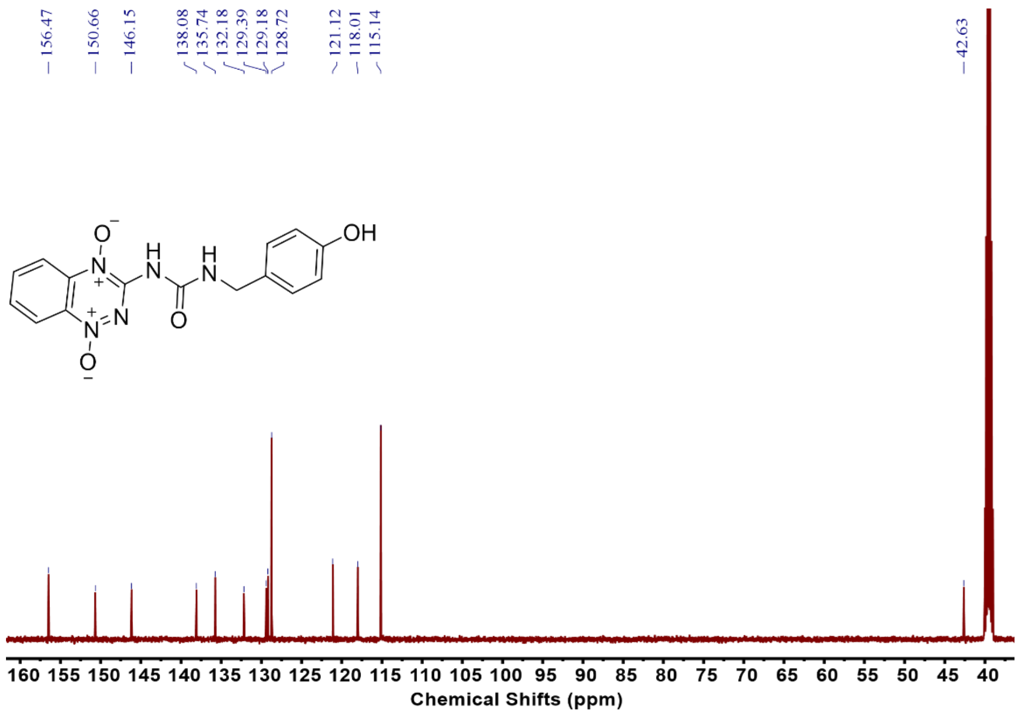

Figure S16. The ^{13}C NMR spectrum of TPZP.

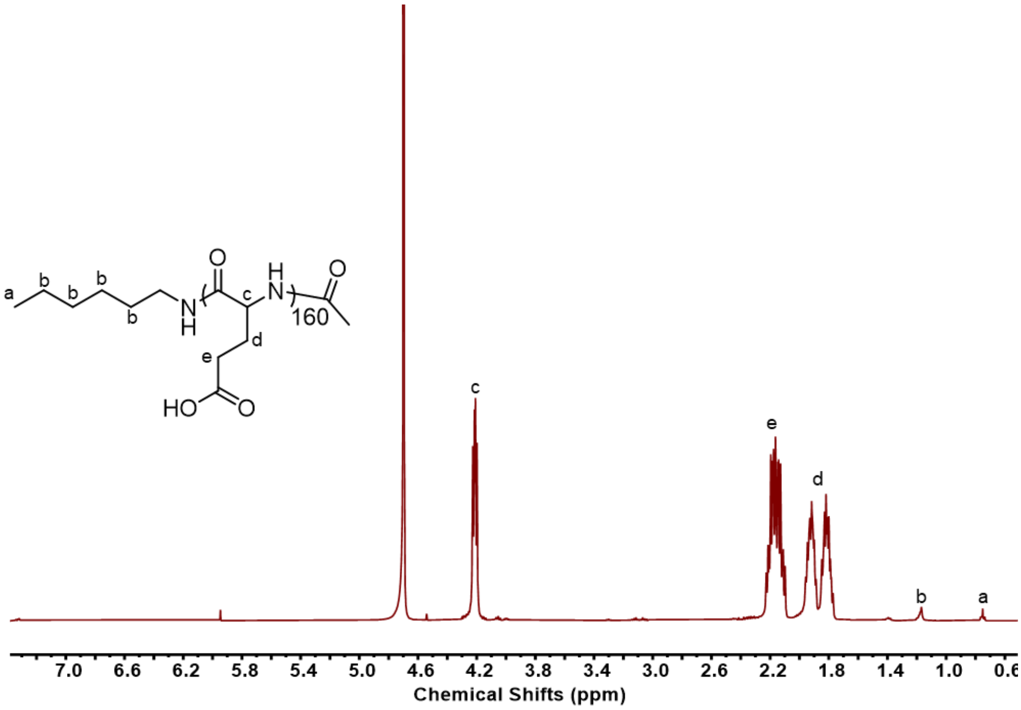

Figure S17. The ^{1}H NMR spectrum of PLG.

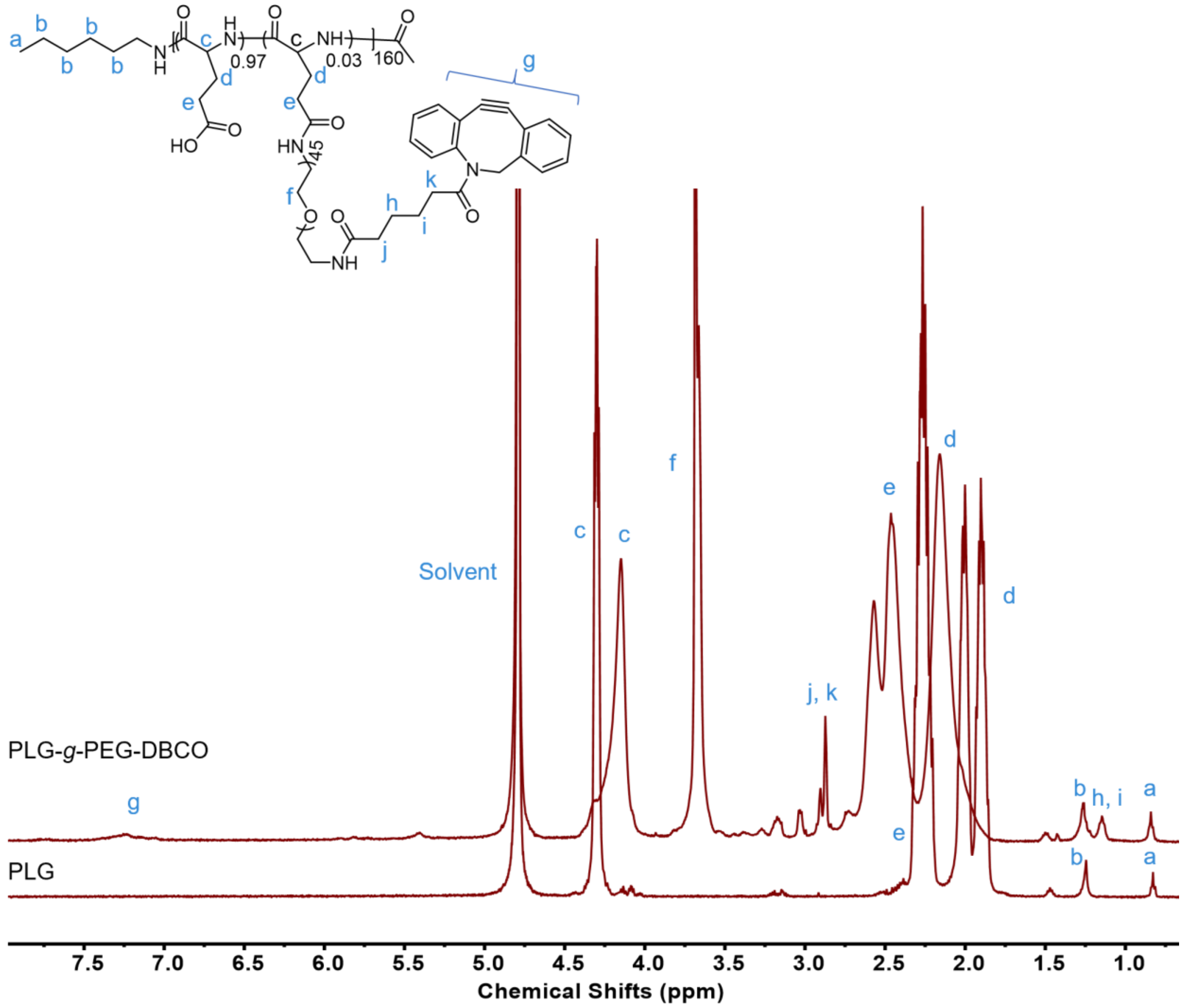

Figure S18. The ^{1}H NMR spectra of PLG and PLG-*g*-PEG-DBCO in D_2O/NaOD.

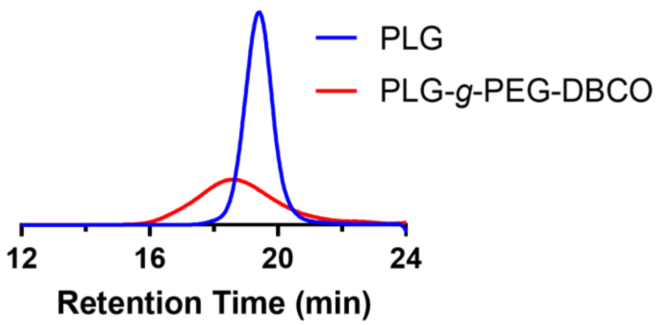

Figure S19. The GPC curves of PLG (Mn = 2.83 $\times 10^3$ g/mol, polydispersity = 1.06) and PLG-*g*-PEG-DBCO (Mn = 3.53 $\times 10^3$ g/mol, polydispersity = 1.42). Using PB (pH = 7.4, 0.2 M) containing 0.1 M $NaNO_3$ as eluent.

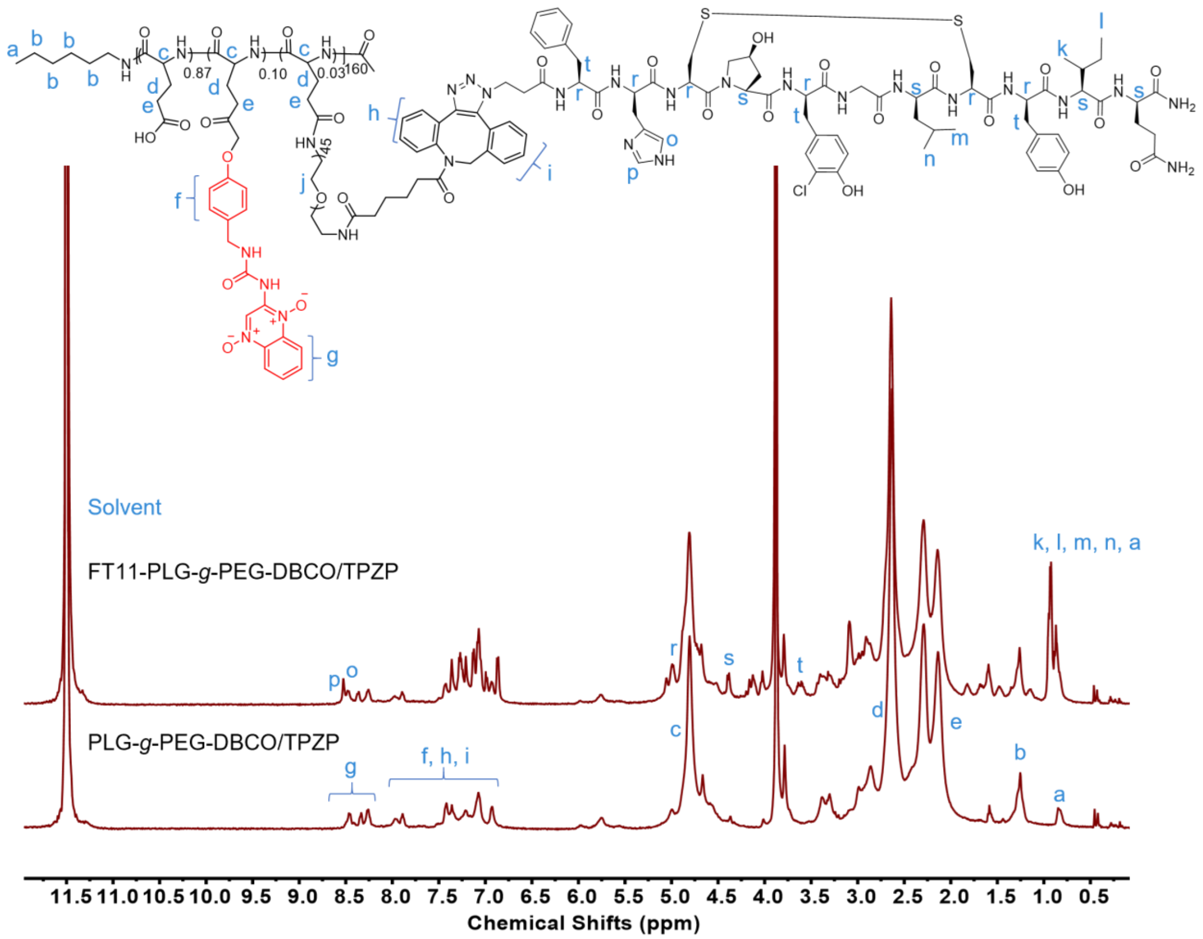

Figure S20. The ^{1}H NMR spectra of PLG-*g*-PEG-DBCO/TPZP

and FT11-PLG-*g*-PEG-DBCO/TPZP in TFA-*d*.

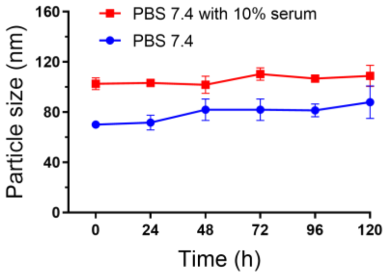

Figure S21. Stability of the FT11-TPZP-NPs tested by DLS, samples were stored in PBS 7.4 solution with or without 10% serum at 4 ℃.

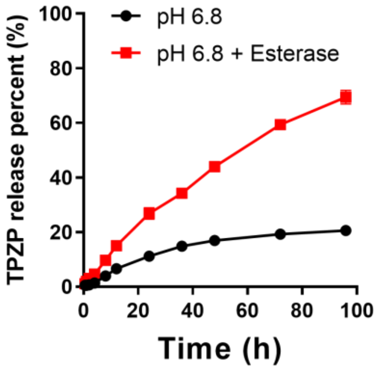

Figure S22. *In vitro* release of TPZP from FT11-TPZP-NPs in phosphate buffered saline at pH 6.8 with or without esterase.

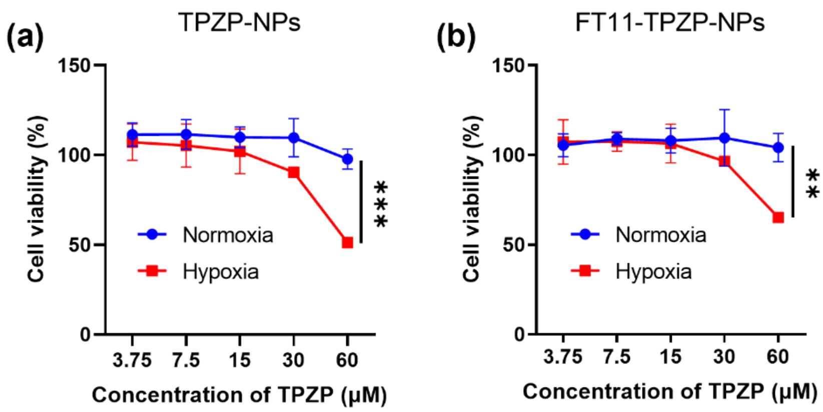

Figure S23. Cell viability of CT26 incubated with different concentration of TPZP-NPs or FT11-TPZP-NPs in normoxia or hypoxia incubated for 24 h (n = 3).

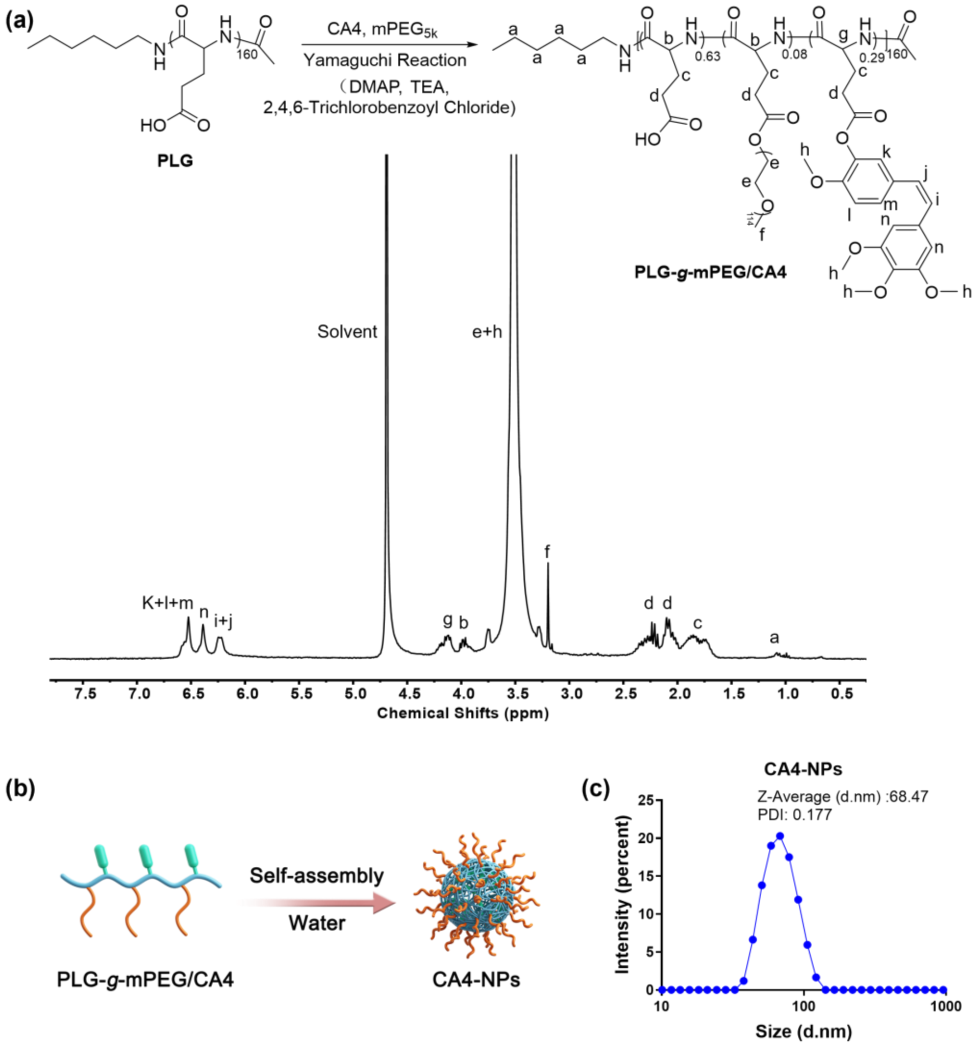

Figure S24. Synthesis and physicochemical characterization of CA4-NPs. (a), Synthesis route and the ^{1}H NMR spectrum of PLG-*g*-mPEG/CA4. (b), PLG-*g*-mPEG/CA4 self-assembly forming CA4-NPs. (c), Size distribution plots of CA4-NPs in PBS (pH = 7.4): Z-Average = 68.47 nm, PDI = 0.177.

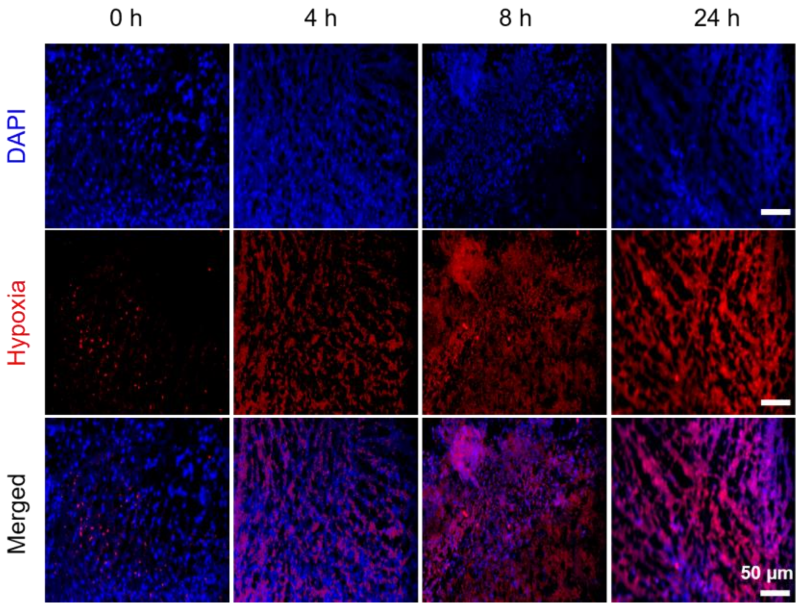

Figure S25. Hypoxia probe (pimonidazole) staining of tumors in mice post CA4-NPs treatment (20 mg/kg, eq to CA4). Scale bar: 20 μm.

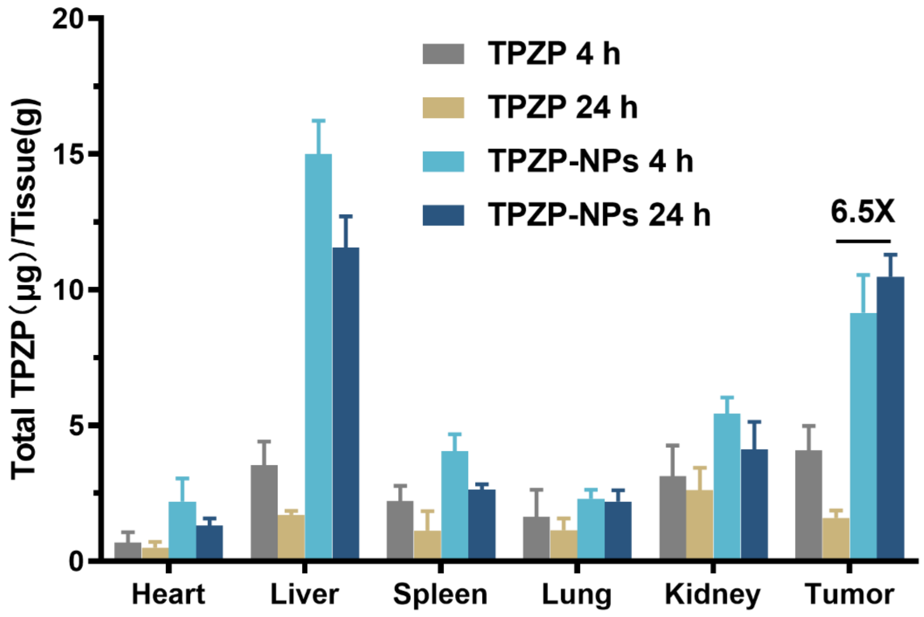

Figure S26. Biodistribution of total TPZP in major organs and tumor of mice after treated with TPZP or TPZP-NPs at different time intervals (n = 3).

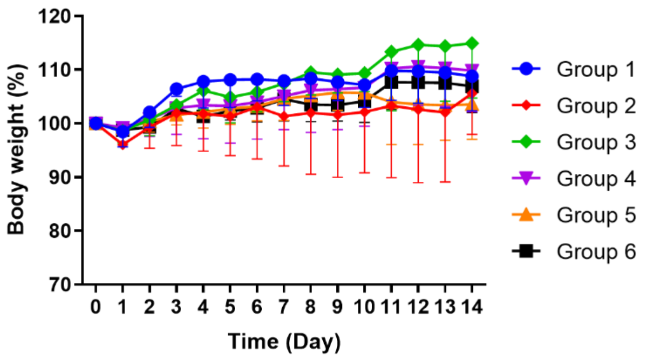

Figure S27. The body weight of mice treated with different dosage of TPZP or TPZP-NPs within 14 days was monitored and recorded (n = 4). Groups: 1) TPZP 10 mg/kg; 2) TPZP 20 mg/kg, one in four mice was dead after treated with TPZP on day 1; 3) TPZP-NPs, 20 mg/kg, eq to TPZP; 4) TPZP-NPs, 40 mg/kg, eq to TPZP; 5) TPZP-NPs, 60 mg/kg, eq to TPZP; 6) TPZP-NPs, 80 mg/kg, eq to TPZP.

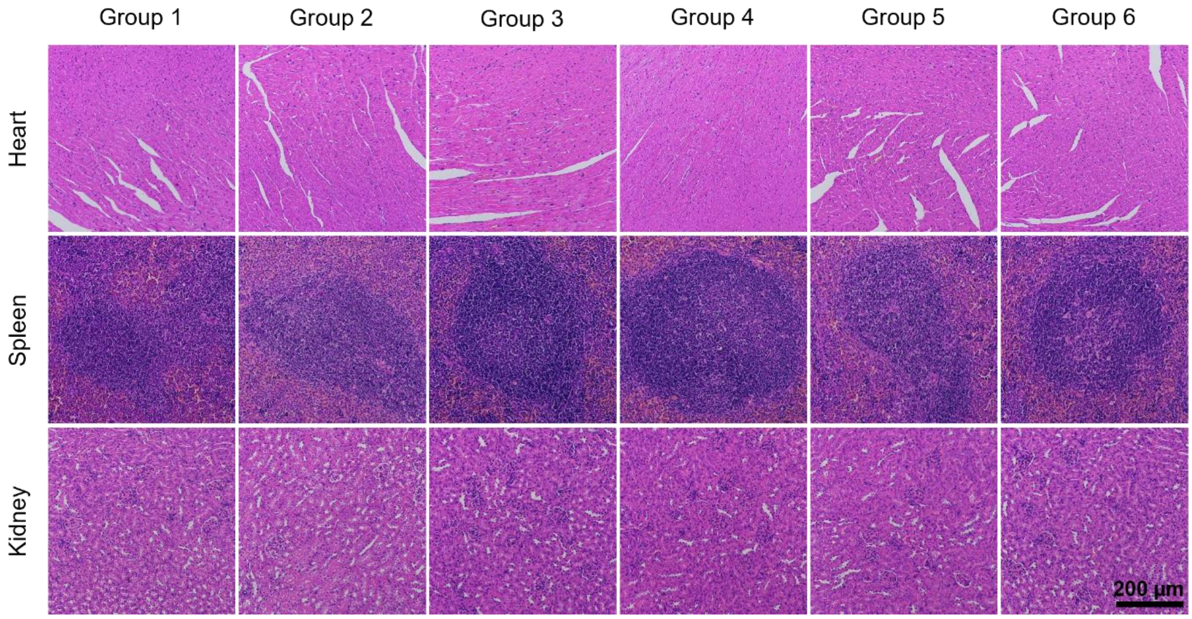

Figure S28. H&E staining of heart, spleen and kidney after different treatment, reflecting the toxicity (scale bar = 200 μm). Groups: 1) PBS; 2) TPZP-NPs; 3) FT11-TPZP-NPs; 4) CA4-NPs; 5) TPZP-NPs + CA4-NPs; 6) FT11-TPZP-NPs + CA4-NPs.

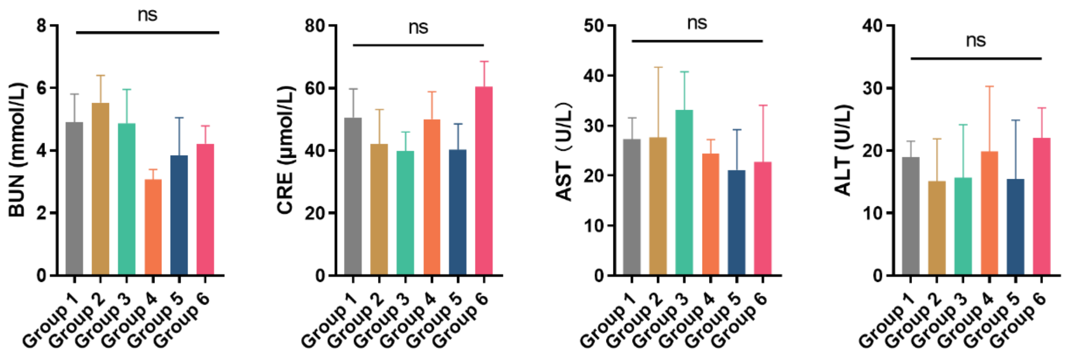

Figure S29. Blood biochemical analysis of CT26 tumor-bearing mice treated with different formulations. Groups: 1) PBS; 2) TPZP-NPs; 3) FT11-TPZP-NPs; 4) CA4-NPs; 5) TPZP-NPs + CA4-NPs; 6) FT11-TPZP-NPs + CA4-NPs.

Table S1. Pharmacokinetic parameters estimated for TPZP, TPZP-NPs and FT11-TPZP-NPs in rats.

**Drugs**	**$t_{1/2}$ [a] (h)**	**AUC_{0-t} [b] (μg/mL·h)**	**MRT_{0-inf_obs} [c] (h)**
TPZP	2.3 ± 0.4	31.5 ± 0.82	3.1 ± 0.6
TPZP-NPs	12.7 ± 3.5	1377.2 ± 98.7	16.5 ± 4.3
FT11-TPZP-NPs	11.8 ± 3.7	1390.6 ± 153.8	14.8 ± 3.6

[a] $t_{1/2}$: half-life.

[b] $AUC_{0–t}$: area under the drug concentration–time curve from 0 to 24 h in plasma.

[c] MRT_{0-inf_obs}: mean residence time.

References

1. Liu, T., Zhang, D., Song, W. *et al.* A poly(l-glutamic acid)-combretastatin A4 conjugate for solid tumor therapy: Markedly improved therapeutic efficiency through its low tissue penetration in solid tumor. *Acta Biomaterialia*. **2017**, *53*, 179-89.
2. Xu, Y., Lv, J., Kong, C. *et al.* A novel hypoxia-activated polymeric Tirapazamine derivative for enhanced antitumor therapy. *Journal of Polymer Science*. **2023**, *61*, 1111-9.
3. Jiang, J., Shen, N., Ci, T. *et al.* Combretastatin A4 Nanodrug-Induced MMP9 Amplification Boosts Tumor-Selective Release of Doxorubicin Prodrug. *Adv. Mater.* **2019**, *31*.
4. Lv, J., Xu, Y., Huang, Y. *et al.* A Novel Vascular Disrupting Agents Noncovalent Polymeric Nanomedicine: Significantly Increased Antitumor Therapeutic Efficiency. *Chinese Journal of Chemistry*. **2022**, *40*, 1447-56.
5. Ma, S., Song, W., Xu, Y. *et al.* A ROS-Responsive Aspirin Polymeric Prodrug for Modulation of Tumor Microenvironment and Cancer Immunotherapy. *CCS Chemistry*. **2020**, *2*, 390-400.
